# Effectively tuning the quantum Griffiths phase by controllable quantum fluctuations

**DOI:** 10.1126/sciadv.adp1402

**Published:** 2024-11-27

**Authors:** Beilin Wang, Guopei Ying, Linhai Guo, Zhiyong Lin, Haiwen Liu, Changgan Zeng

**Affiliations:** ^1^ CAS Key Laboratory of Strongly-Coupled Quantum Matter Physics, and Department of Physics, University of Science and Technology of China, Hefei 230026, China.; ^2^ International Center for Quantum Design of Functional Materials (ICQD), Hefei National Research Center for Physical Sciences at the Microscale, University of Science and Technology of China, Hefei 230026, China.; ^3^ Hefei National Laboratory, University of Science and Technology of China, Hefei 230088, China.; ^4^ Center for Advanced Quantum Studies, Department of Physics, Beijing Normal University, Beijing 100875, China.; ^5^ Key Laboratory of Multiscale Spin Physics, Ministry of Education, Beijing Normal University, Beijing 100875, China.; ^6^ Interdisciplinary Center for Theoretical Physics and Information Sciences, Fudan University, Shanghai 200433, China.

## Abstract

Quantum Griffiths phase (QGP), marked by a quantum Griffiths singularity with a divergent effective critical exponent, has garnered considerable attention in the realm of superconductivity. However, the ability to control QGP remains elusive. Here, we demonstrate that QGP at the LaAlO_3_/KTaO_3_(110) interface can be efficiently modulated by the orientation of applied magnetic field: With a perpendicular field, an anomalous QGP emerges in the low-temperature regime, characterized by a decreasing critical field as temperature lowers; conversely, with a parallel field, a normal QGP arises, where the critical field increases with decreasing temperature. Such opposite characteristics stem from the controllable quantum fluctuations and conductivity corrections under distinct magnetic field orientations. Furthermore, we show the effective tuning of the phase boundary by electrostatic gating, attributed to the gate-controlled quantum fluctuations. These findings not only demonstrate how to experimentally manipulate QGP but also provide a comprehensive understanding of how quantum fluctuations can effectively modulate QGP.

## Teaser

Quantum Griffiths phase is controlled by adjusting quantum fluctuations via magnetic field orientation and gate voltage changes.

## INTRODUCTION

Quantum phase transitions (QPTs), driven by quantum fluctuations, lie at the forefront of scientific exploration for their crucial role in unraveling intricate quantum states and advancing quantum device fabrication (*1*–*6*). Disordered two-dimensional (2D) superconductors have emerged as a prominent platform for studying QPTs owing to their enhanced quantum fluctuations (*7*, *8*). The interplay between quantum fluctuations and dissipation substantially enriches the physics of QPTs, yielding novel quantum phenomena in 2D superconducting systems, such as the quantum metal state (*4*, *6*, *9*–*21*) and the quantum Griffiths phase (QGP) (*17*, *22*–*29*). The emergence of QGP in 2D superconductors is attributed to the presence of disorder-induced spatially rare superconducting regions embedded within the normal state (*30*, *31*). As the temperature approaches zero, these superconducting regions grow, and their slow dynamics result in a QGP, characterized by a divergent effective critical exponent (*z*ν) known as the quantum Griffiths singularity (QGS), which fundamentally differs from QPT in clean systems (*30*–*34*). Research on QGP has garnered substantial attention, becoming a crucial element of 2D superconductivity studies (*35*, *36*).

QGP has been observed in various 2D superconductors so far (*22*–*25*, *27*–*29*), typically exhibiting a normal phase boundary where the critical field increases monotonically with decreasing temperature. Theoretical studies have indicated that superconducting fluctuations dominate the conductivity correction near the quantum critical point (*37*–*41*). A recent experimental study reveals that superconducting fluctuations with strong spin-orbit coupling (SOC) can result in a novel type of QGS, known as anomalous QGS (*26*). This is characterized by a QGP displaying an anomalous phase boundary under a perpendicular magnetic field, where the critical field decreases with decreasing temperature near the quantum critical point. While numerous studies have reported observations of QGP in various materials (*22*–*29*), the ability to efficiently manipulate this exotic state has remained a challenge. Here, we have discovered both anomalous and normal QGPs within one system, leveraging the newly discovered (110)-oriented LaAlO_3_/KTaO_3_ (LAO/KTO) superconducting interface (*42*). Furthermore, we present an effective strategy to manipulate QGP through the controlled induction of quantum fluctuations, facilitated by changing the orientation of the magnetic field and gate voltage.

## RESULTS

### Quantum metal state at the LAO/KTO(110) interface

**Fig. 1.**
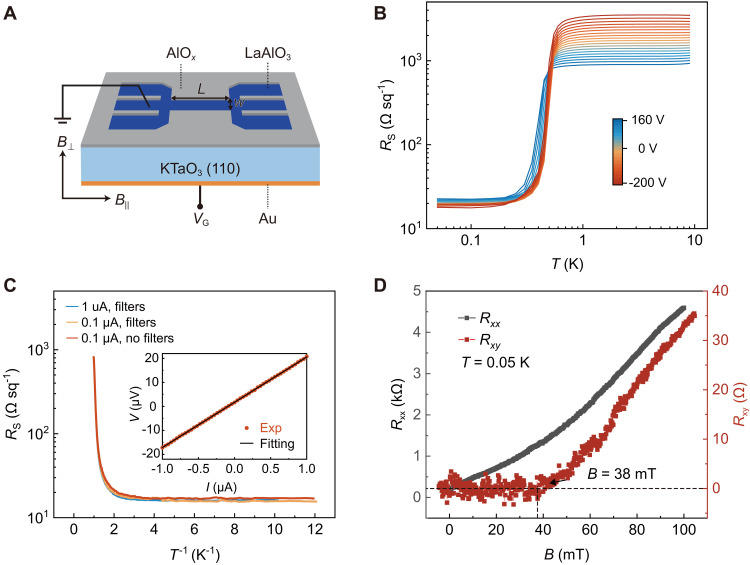
Characterization of the LAO/KTO(110) interface. (**A**) Schematic of the LAO/KTO(110) interface with a standard Hall bar used for resistance measurements. (**B**) Temperature dependence of the sheet resistance *R*_S_(*T*) of sample #1 for different gate voltages (*V*_G_). (**C**) Arrhenius plot of the sheet resistance with and without resistor-capacitor filters for different currents at *V*_G_ = −200 V. Inset: A typical linear *I*-*V* curve in the low current range at *V*_G_ = −200 V. (**D**) Magnetic field dependence of the longitudinal resistance (*R_xx_*) and Hall resistance (*R_xy_*) for *V*_G_ = 0 V of sample #2. The intersection of black dashed lines indicates the maximum magnetic field with zero Hall resistance.

We fabricated Hall bar devices for the LAO/KTO(110) interface, as schematically illustrated in Fig. 1A (see Materials and Methods for details). Uniformly amorphous LAO films with a nominal thickness of 10 nm exhibit a smooth surface with a root-mean-square roughness of ~0.1 nm (see fig. S2). Figure 1B shows the temperature-dependent sheet resistance *R*_S_(*T*) of the LAO/KTO(110) interface for various gate voltages (*V*_G_), revealing *V*_G_-dependent superconducting transitions. The superconducting transition temperature *T*_C_ is 0.76 K for *V*_G_ = −200 V, defined at 90% of the normal-state resistance (for detailed discussion, see text S1). The nonlinear current (*I*)–voltage (*V*) response, described by the *V* ∝ *I*^3^ dependence, and the *R*_S_(*T*) characteristics, expressed as *R*_S_(*T*) ∝ exp [−*b*(*T*/*T*_BKT_ − 1)^−1/2^] (where *b* is the material parameter), collectively signify a typical Berezinskii-Kosterlitz-Thouless transition (see fig. S3 for details), supporting the 2D nature of the superconducting transition.

Notably, the quantum metal state emerges across the entire accessible range of *V*_G_ as the temperature decreases. Specifically, *R*_S_(*T*) rapidly drops and saturates at a nonzero value that is about two orders of magnitude smaller than its normal-state resistance. The plateau-like residual resistance below 0.3 K suggests the presence of a quantum metal state (*4*, *6*, *9*–*21*). Similar features are observed in sample #2 (see fig. S4). To gain deeper insight into the nature of the residual resistance, we measured the *I*-*V* curve at a temperature of 0.05 K. The *I*-*V* curve exhibits a linear relationship below 1 μA, confirming that the measurements are within the ohmic regime and ruling out the influence of Joule heating (inset of Fig. 1C) (*19*). Moreover, the residual resistance survives in measurements with resistor-capacitor filters (Fig. 1C), excluding the origin from radiation thermalization (*19*) (see Materials and Methods for details). Notably, the vanishing Hall resistance below 38 mT shown in Fig. 1D reveals the presence of particle-hole symmetry (*15*, *19*, *21*). The collective evidence from these observations unambiguously points to the existence of quantum metal in our systems.

Oxide interfaces, such as the LAO/SrTiO_3_(001) interface (*16*, *21*) and the LAO/KTO(111) interface (*6*, *43*), can exhibit a superconducting state or a quantum metal state in the absence of a magnetic field, depending on the degree of disorder (*44*). In the presence of pronounced disorder, superconductivity is locally disrupted, and induced fermionic metal states manifest as weak Josephson links between the superconducting puddles (*16*, *21*). Consequently, the existence of weak Josephson links induces pronounced phase fluctuations and dissipative transport between distinct superconducting puddles, ultimately resulting in the quantum metal state. For the LAO/KTO(110) interface adopted in this study, our observations reveal a quantum metal state, while a previous investigation identified a superconducting state (*42*). This difference originates from the stronger disorder in our systems stemming from variations in sample fabrication (*44*). This is supported by the mobility comparison. The mobility of our sample, estimated at 43 cm^2^ V^−1^ s^−1^ (fig. S4C), is substantially lower than the value of 81 cm^2^ V^−1^ s^−1^ reported in the previous study (*42*).

### Anomalous QGP induced by the perpendicular magnetic field

Previously, QGP has been widely observed in various 2D superconductors (*22*–*29*). Recent studies on ion-gated ZrNCl and MoS_2_ have unveiled a phase diagram demonstrating that QGP not only manifests during a superconductor-metal transition but also can coexist with a quantum metal state under a magnetic field (*17*). In this case, the quantum metal state consists of superconducting puddles (with a relatively large value of order parameter) and their surrounding dissipative weak Josephson links. When the applied magnetic field approaches the quantum critical point, superconducting rare regions (with a marginal value of order parameter) emerge due to the quenched disorder effect, giving rise to QGP. Here, we also observe the coexistence of QGP with a quantum metal state at the LAO/KTO(110) interface, as elaborated below.

**Fig. 2.**
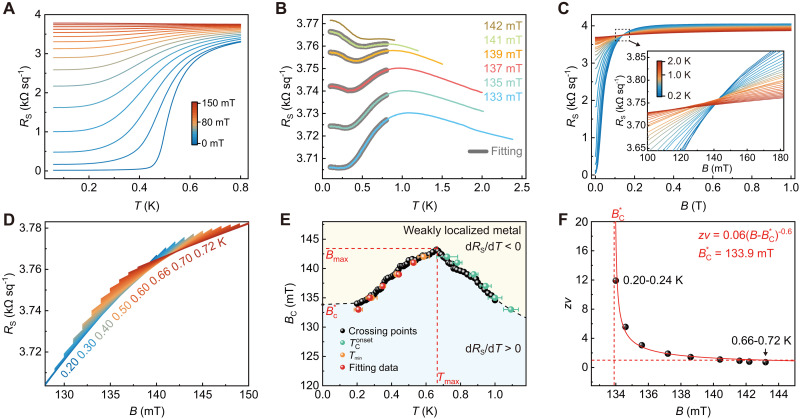
Anomalous QGS at the LAO/KTO(110) interface under the perpendicular magnetic field. (**A**) *R*_S_(*T*) curves of sample #1 measured at different perpendicular magnetic fields. (**B**) *R*_S_(*T*) curves measured at various magnetic fields from 133 to 142 mT exhibit reentrant behavior. Bold gray lines show the *R*_S_(*T*) curves fitted with the superconducting fluctuation theory. (**C**) *R*_S_(*B*) curves measured at different temperatures. Inset: A zoomed-in view of the magnetoresistivity near the crossing region in (C). (**D**) *R*_S_(*B*) curves measured for temperatures ranging from 0.20 to 0.72 K in 0.01-K steps. (**E**) Crossing points *B*_C_(*T*) obtained from neighboring *R*_S_(*B*) curves (black points), *T*_min_ (orange points) and onset $T_{C}^{\text{onset}}$ (green points) derived from *R*_S_(*T*) curves, determine the phase boundary of the QPT, which quantitatively matches with the fitting data (red points) obtained from *R*_S_(*T*) curves fitted with the superconducting fluctuation theory. Error bars represent the uncertainty of data defined by d*R*_S_/d*T* = 0 due to experimental resolution. (**F**) Critical exponent *z*ν as a function of the perpendicular magnetic field follows well the activated scaling law with $B_{C}^{*}$ = 133.9 mT, indicating the existence of anomalous QGS. All measurements were performed in sample #1 at *V*_G_ = −200 V.

Figure 2A shows that the quantum metal can be modulated by the perpendicular magnetic field, undergoing a QPT into a weakly localized metal with a critical resistance below the quantum resistance *h*/4*e*^2^, where *h* is the Planck constant and *e* is the elementary charge. Moreover, the remarkable Hall signal (fig. S5A) also suggests a pair-breaking–induced QPT (*22*). Remarkably, a reentrant behavior of *R*_S_(*T*) occurs when the field slightly exceeds 130 mT (see Fig. 2B). In a conventional superconductor-metal transition, the phase boundary—the borderline separating the regions of d*R*_S_/d*T* < 0 and d*R*_S_/d*T* > 0—demonstrates a monotonic feature. In sharp contrast, here we find that the resistivity *R*_S_(*T*) exhibits reentrant behavior: *R*_S_(*T*) initially decreases with falling temperature and reaches a minimum at *T*_min_; further cooling beyond *T*_min_ triggers a reversal with *R*_S_(*T*) ascending. This reentrant behavior gives rise to the anomalous QGP with a nonmonotonic phase boundary.

We further investigated the QPT behavior by measuring magnetoresistance at various temperatures (Fig. 2C). Systematic measurements in the low-temperature regime are presented in Fig. 2D. The crossing points *B*_C_(*T*) extracted from the neighboring *R*_S_(*B*) curves in Fig. 2D determine the phase boundary of the QPT (see Fig. 2E, black points). Upon closer examination of the crossing region (Fig. 2C, inset), it is evident that the crossing points shift with temperature, deviating from the conventional QPT scenario (*1*, *2*). Specifically, *B*_C_ initially increases with decreasing temperature, reaching a maximum *B*_max_ of 143.2 mT at *T*_max_ of 0.66 K (Fig. 2E, black points). Further cooling leads to a decrease in *B*_C_, resulting in nonmonotonic behavior for *B*_C_ and an anomalous phase boundary below 0.66 K. Determining the phase boundary (d*R*_S_/d*T* = 0) by the minima or maxima of the *R*_S_(*T*) curves is also a commonly used method (*17*, *22*–*24*, *26*, *45*). We also plotted $T_{C}^{\text{onset}}$ (green points) and *T*_min_ (orange points) in Fig. 2E. Here, $T_{C}^{\text{onset}}$ represents the emergence of superconducting fluctuations and is the maximum of the *R*_S_(*T*) curve from Fig. 2B. The phase boundary determined by crossing points aligns well with $T_{C}^{\text{onset}}$ (green points) and *T*_min_, which suggests the reliability of these extracted crossing points.

We next conducted the finite-size scaling analysis, validated by numerous studies (*17*, *22*–*29*), on the *R*_S_(*B*) curves to determine the critical behavior of this anomalous QPT. To ensure the robustness of our scaling analysis, we have taken careful steps in both measurements and data processing (see text S2 for details). Generally, the *R*_S_(*B*) curves at neighboring temperatures are grouped together so that the critical transition region can be proximately regarded as a single critical point (*B*_C_, *R*_C_) (*2*, *22*). *R*_S_(*B*) takes the scaling form (*2*, *22*): $R_{s}\left(B,T\right)=R_{c}\cdot F\left(|B-B_{c}|T^{-\frac{1}{z\nu }}\right)$, where *F* is an arbitrary function with *F*(0) = 1, *z* is the dynamical critical exponent, and ν is the correlation length exponent. We then plotted the scaling curves of *R*_S_(*B*)/*R*_C_ at various temperatures against the scaling variable*t*∣*B* − *B*_C_∣. Here, *t* = (*T*/*T*_0_)^−1/*z*ν^, obtained by minimizing the numerical difference between the *R*(*B*, *t*) at a particular temperature *T* and the *R*_S_(*B*, *t* = 1) at the lowest temperature *T*_0_. The effective critical exponent *z*ν is obtained from a linear fitting between ln*T* and ln*t* (see figs. S6 to S9 for details).

Figure 2F demonstrates that *z*ν varies with the magnetic field and follows the activated scaling law $z\nu \propto {|B-B_{C}^{*}|}^{-0.6}$, indicating the existence of QGS (*46*, *47*). Notably, the effective critical exponent *z*ν diverges upon approach to the infinite-randomness quantum critical point $B_{C}^{*}$ = 133.9 mT from the high-field side (*B* > $B_{C}^{*}$). This behavior differs distinctly from the normal QGS, where *z*ν diverges as $B_{C}^{*}$ is approached from the low-field side (*B* < $B_{C}^{*}$) (*17*, *22*–*25*, *27*–*29*). The unique critical behavior observed here is referred to as anomalous QGS (*26*), distinguished by a QGP with an anomalous boundary existing in the regime (*T*_max_, Δ*B*). Here, *T*_max_ and $\Delta B=B_{\text{max}}-B_{C}^{*}$ correspond to the temperature and magnetic field ranges of the anomalous phase boundary, respectively (refer to Fig. 2E). The generality of these features is validated in other samples as well (see fig. S10). To note, while we have identified the coexistence of the quantum metal state and QGP, they remain independent phenomena (see text S3), as corroborated by the observation of QGP in various 2D superconductors that lack the quantum metal state (*22*–*29*).

The quantum dynamics of QPT induced by the perpendicular field also demonstrate exotic features. The critical exponent *z*ν reaches up to 12 between 0.20 to 0.24 K (see Fig. 2F), uncovering the ultraslow dynamics and strong dissipation effect of the system (*31*, *34*). In addition to the large value of *z*ν, the enhanced dissipation effect of the LAO/KTO(110) interface under a perpendicular magnetic field also drastically changes the dynamics near zero temperature. Below 0.2 K, the magnetoresistance curves across different temperatures converge (refer to fig. S5B), rendering it challenging to conduct scaling analysis to determine *z*ν. These observations are reminiscent of the characteristics of a smeared phase transition, where sufficiently strong ohmic dissipation tends to freeze the dynamics of individual rare regions and gives rise to a local static order independent of the bulk (*31*, *34*, *48*). Thus, the strength of ohmic dissipation may account for the differences between our work and the previous study on Pb films (*26*). These intriguing dynamical features of LAO/KTO(110) under a perpendicular field require a deep exploration of the interplay between dissipation process and magnetic field, and will be pursued in future investigations.

We next delve into the origin of the anomalous phase boundary of QGP under a perpendicular magnetic field. After careful analysis, we have ruled out several possible mechanisms, including the Werthamer-Helfand-Hohenberg scenario, the competition between antiferromagnetism and superconductivity, and the competition between Josephson coupling and Coulomb interaction (see text S4 for details). Previous theoretical investigations have revealed that four types of quantum fluctuations contribute to conductivity corrections around the quantum critical point, enabling a quantitative analysis of the phase boundary under the perpendicular magnetic field (*37*–*41*). These corrections can be classified into two groups: terms not influenced by spin-orbit scattering (the Aslamazov-Larkin term, the Maki-Thompson term, and the density of state term) and terms influenced by spin-orbit scattering [the diffusion coefficient renormalization (DCR) term]. The first three terms typically lead to an upward curvature of *B*_C_(*T*) (*49*), exhibiting a normal phase boundary (*22*), in sharp contrast to the observed anomalous phase boundary (Fig. 2E). On the other hand, the sign of the DCR term is undetermined and is substantially influenced by spin-orbit scattering (*40*), which may result in the emergence of an anomalous phase boundary.

Specifically, strong SOC scattering can introduce additional spin-triplet channels in the Cooperon propagator (*50*) and change the sign of the DCR term. The change is a prerequisite for the crossover of the phase boundary from an upward curvature to a downward curvature, reminiscent of the weak/anti-weak localization crossover under the influence of pronounced spin-orbit scattering (*50*). Previous investigations have demonstrated that the KTO(110) heterointerfaces have large SOC energy (*51*, *52*). Thus, strong SOC at the LAO/KTO(110) interface induces different conductivity correction coefficients with opposite signs compared to the previous theory (*38*) (see text S5 for details). This results in a downward curvature of *B*_C_(*T*) with reentrant behavior under the perpendicular magnetic field, which is validated by the experimental results depicted in Fig. 2 (B and E). According to the combinational effect of strong SOC scattering and superconducting fluctuations, we quantitatively reproduced the reentrant behavior (Fig. 2B and details in text S5). The minima of the fitted *R*_S_(*T*) curves are consistent with *T*_min_, as summarized in Fig. 2E. As the system approaches the quantum critical point along this anomalous phase boundary, the importance of the DCR term becomes evident in the emergent rare region that dominates the critical behavior of the system, leading to anomalous QGS. Notably, this conductivity correction from quantum fluctuations only needs the fluctuating spin-triplet Cooperon channels but not requires the formation of spin-triplet Cooper pairs into a coherent Bose-Einstein condensation state (also see text S5).

### Normal QGP induced by the parallel magnetic field

**Fig. 3.**
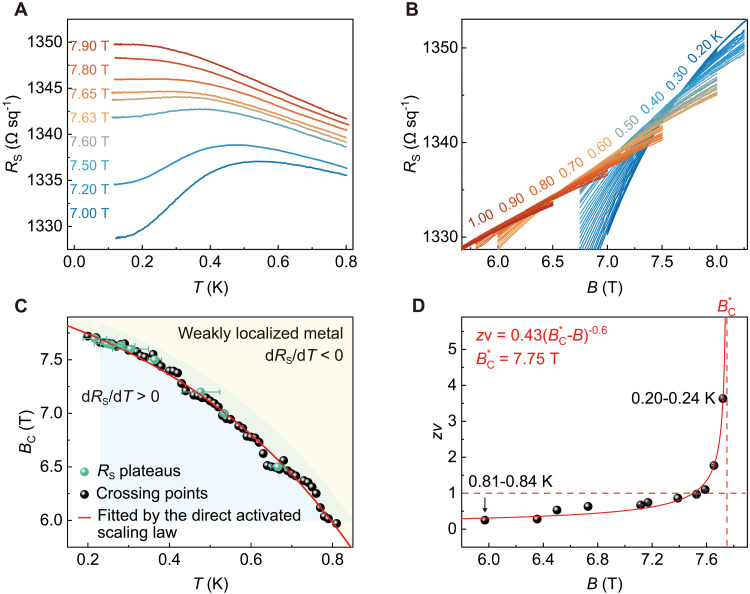
Normal QGS at the LAO/KTO(110) interface under the parallel magnetic field. (**A**) *R*_S_(*T*) curves measured at different parallel magnetic fields from 7.00 to 7.90 T show no signs of reentrant behavior. (**B**) *R*_S_(*B*) curves measured at the temperature ranging from 0.20 to 1.00 K in 0.01-K steps. (**C**) Crossing points *B*_C_(*T*) obtained from neighboring *R*_S_(*B*) curves (black points) and resistance plateaus (green points) from *R*_S_(*T*) curves in (B) are well fitted with the direct activated scaling analysis with irrelevant correction (see text S6 for details). Error bars represent the uncertainty of data defined by d*R*_S_/d*T* = 0 due to experimental resolution. (**D**) Critical exponent *z*ν as a function of the parallel magnetic field follows well the activated scaling law with $B_{C}^{*}$ = 7.75 T, indicating the existence of QGS. All measurements were performed in sample #1 at *V*_G_ = 0 V.

In stark contrast to the case with a perpendicular field, the reentrant behavior of the *R*_S_(*T*) curves disappears under parallel magnetic fields (Fig. 3A). Furthermore, the crossing points *B*_C_(*T*) (see Fig. 3C), extracted from neighboring *R*_S_(*B*) curves in Fig. 3B, decrease monotonically with increasing temperature. The phase boundary determined by these crossing points is evidently different from the perpendicular field case but resembles those of the normal QGP (*17*, *22*–*25*, *27*–*29*). After conducting the finite-size scaling analysis to determine *z*ν (see figs. S12 to S14 for details), we find that *z*ν is well described by the activated scaling law $z\nu \propto {|B-B_{C}^{*}|}^{-0.6}$ with $B_{C}^{*}$ = 7.75 T (see Fig. 3D), providing evidence of QGS (*46*, *47*). Such features are also reproduced in sample #2 (fig. S15). Additionally, direct evidence of QGS is provided by the direct activated scaling analysis (*28*, *29*) (see text S6 and fig. S16 for details).

To date, QGS under a parallel magnetic field has only been reported in PbTe_2_ thin films (*28*) and β-W thin films (*29*). Under a parallel magnetic field, the dominant contribution comes from the Zeeman effect (*53*–*55*). While the Zeeman splitting disrupts the coherence of some Cooper pairs, large rare regions of superconducting puddles persist in the critical region at low temperatures due to quenched disorder. This leads to the emergence of QGP, thereby giving rise to QGS (*28*, *29*). In contrast to the case under a perpendicular magnetic field, the boundary of QGP under the parallel field (see Fig. 3C) demonstrates normal behavior with no discernible curvature in the low-temperature regime.

### *V*_G_ modulation of the anomalous phase boundary of QGP and SOC

**Fig. 4.**
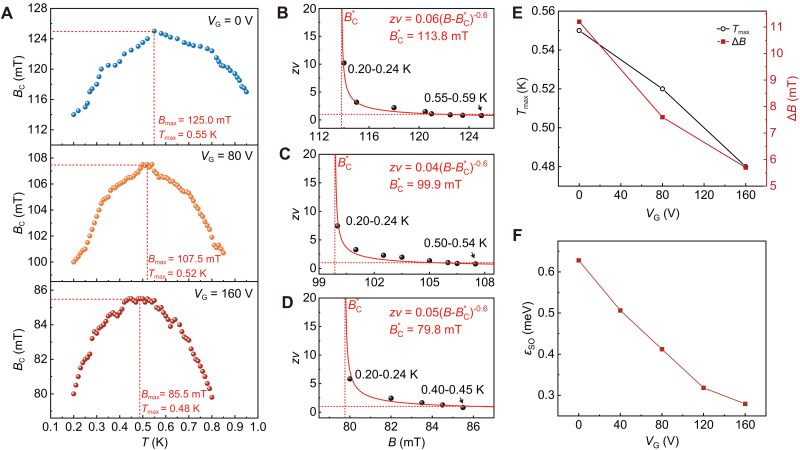
*V*_G_ modulation of the anomalous phase boundary and spin-orbit coupling energy. (**A**) Crossing points *B*_C_(*T*) obtained from neighboring *R*_S_(*B*) curves for *V*_G_ = 0, 80, and 160 V. (**B** to **D**) Critical exponent *z*ν as a function of the perpendicular magnetic field for *V*_G_ = 0, 80, and 160 V. All follow the activated scaling law $z\nu \propto {|B-B_{C}^{*}|}^{-0.6}$ (red solid lines). (**E**) *T*_max_ and Δ*B* as a function of *V*_G_, where *T*_max_ and $\Delta B=B_{\text{max}}-B_{C}^{*}$ represent the temperature and magnetic field ranges of the anomalous phase boundary, respectively (refer to Fig. 2E). (**F**) SOC energy ε_SO_ as a function of *V*_G_. All measurements were performed in sample #3.

Strong SOC scattering can alter the sign of the DCR term (*26*) and thus enable the crossover of the phase boundary from an upward curvature to a downward curvature. This implies that the anomalous phase boundary of QGP under the perpendicular magnetic field can be tuned by adjusting SOC. The remarkable tunability of SOC through gating in our system (*51*) provides an opportunity to investigate the intractable mechanism of the anomalous QGP. We thus explore the critical behavior at different gate voltages. Figure 4A shows the crossing points *B*_C_ obtained from neighboring *R*_S_(*B*) curves at three distinct gate voltages (*V*_G_ = 0, 80, and 160 V) of sample #3, indicating the presence of an anomalous phase boundary. Figure 4 (B to D) demonstrates that *zν* follows the activated scaling law $z\nu \propto {|B-B_{C}^{*}|}^{-0.6}$, confirming the existence of anomalous QGS with different $B_{C}^{*}$ at these gate voltages. The value of *z*ν (0.20 to 0.24 K) decreases as *V*_G_ increases from 0 to 160 V, indicating a relatively weaker effect of dissipation (*31*, *34*). Furthermore, the relationships between *T*_max_, $\Delta B=B_{\text{max}}-B_{C}^{*}$, and *V*_G_ show a clear decreasing trend in the anomalous phase boundary with increasing *V*_G_ (see Fig. 4E). Notably, the field range (Δ*B*) can be tuned by ~50% of its initial value as *V*_G_ varies from 0 to 160 V.

To elucidate the *V*_G_ dependence of SOC strength, we adopted a well-validated spin-orbit scattering model (*51*, *56*, *57*). This model predicts an enhancement of the parallel upper critical field *B*_C∥_ due to strong SOC scattering, expressed as $B_{C∥}=0.602\sqrt{\varepsilon_{\text{SO}}/k_{B}T_{C}}B_{P}$ (*51*, *56*, *57*), where $\varepsilon_{\text{SO}}=\hbar /\tau_{\text{SO}}$, and τ_SO_ is the spin-orbit scattering time. The Pauli limiting field $B_{P}=1.76k_{B}T_{C}/\sqrt{2}\mu_{B}$, where μ_B_ is the Bohr magneton. As shown in Fig. 4F, our estimation of SOC energy ε_SO_ (details shown in fig. S17) reveals a decreasing trend with increasing *V*_G_, consistent with the previous study (*51*). Clearly, this underscores a robust correlation between SOC energy and the anomalous phase boundary dependence on *V*_G_. Similar *V*_G_ modulation of the anomalous QGP boundary and SOC strength are observed in sample #4 (see fig. S18).

For systems without the SOC effect, quenched disorder can enhance the critical field of 2D superconductors due to weakly Josephson-coupled superconducting islands (*49*). This enhancement gives rise to a normal boundary of QGP (*22*). In contrast, strong SOC scattering may introduce a minus sign of Josephson coupling between the superconducting islands (*58*), which can diminish the critical field. From the aspect of conductivity correction, large SOC reverses the sign of the DCR term conductivity corrections (*40*, *41*), leading to an anomalous boundary of QGP around the quantum critical point (22). As SOC strength decreases, the influence of DCR corrections becomes marginal, resulting in a more flattened downward curvature of *B*_C_(*T*). Meanwhile, the gradual marginalization of dissipation is also indicated by the decrease of *zν* (*31*, *34*). Thus, the temperature and magnetic field ranges of the anomalous boundary decrease, as illustrated in Fig. 4E. By further reducing SOC strength, the anomalous phase boundary is expected to disappear under a perpendicular magnetic field.

## DISCUSSION

The quantum fluctuations near the quantum critical point under a perpendicular magnetic field are theoretically revealed through clusters of coherently fluctuating Cooper pairs (*40*, *41*). These clusters may be associated with the precursor formation of rare regions (large superconducting islands) (*26*). The fluctuating Cooper pairs rotate around a fixed center and contribute to longitudinal charge transport only when the temperature deviates from zero (*40*, *41*). In this situation, the effect of quantum fluctuations on conductivity correction is primarily determined by the renormalization of the single-particle diffusion coefficient, namely, the DCR term. The strong SOC introduces spin-triplet channels in the Cooperon propagator (*50*), substantially changing the quantitative contribution of the DCR term (*26*). This change leads to different conductivity correction coefficients with opposite signs compared to those from the previous theory (*38*) (see text S5 for details). In other words, the strong spin-orbit scattering induces destructive interference between the superconducting islands (*58*), leading to the destruction of superconductivity.

Therefore, at the LAO/KTO(110) interface, quantum fluctuations with strong SOC exert a remarkable influence on the conductivity near the quantum critical point and result in a decreased critical field with decreasing temperature. As a result, QGP manifests an anomalous phase boundary shown in Fig. 2E. This anomalous phase boundary can be modulated through the quantum fluctuations influenced by tunable spin-orbit scattering (Fig. 4). Additionally, the relatively strong disorder (see text S7 for details) both gives rise to the quantum metal state (Fig. 1B) and large conductivity corrections due to enhanced quantum fluctuations. These large conductivity corrections are responsible for the emergence of anomalous QGP (*26*). Under a parallel magnetic field, the dominant contribution arises from the Zeeman effect (*53*–*55*), and thus, the Cooperon propagator is not largely influenced by the magnetic field strength (*40*, *41*). Consequently, the conductivity corrections from quantum fluctuations are negligible, yielding a normal phase boundary without reentrant features, as presented in Fig. 3 (A and C).

In summary, we have demonstrated that the LAO/KTO(110) interface hosts both QGP and the quantum metal state. We experimentally observed anomalous QGP and normal QGP within one system, and demonstrated that these phases can be efficiently modulated through magnetic field orientation and gating. Such effective tuning of QGP is attributed to varying conductivity corrections near the quantum critical point, resulting from controllable quantum fluctuations with strong SOC by magnetic field orientation and gate voltage. The magnetic field orientation– and gate-tunable QGPs advance the present understanding of the impact of quantum fluctuations on QGP and offer insights for the exploration of exotic QPTs through controllable quantum fluctuations.

## MATERIALS AND METHODS

### Device fabrication

The fabrication process for Hall bars is illustrated in fig. S1. To protect the LAO/KTO(110) interface, we began by depositing 2-nm-thick LAO films on KTO(110) single-crystalline substrates (MTI Corporation) using the pulsed laser deposition (PLD) method. This thickness remains below the critical threshold (*d*_C_) required for conducting interfaces (*59*). Then, a positive resist, polymethyl methacrylate (PMMA 450 K), was spun to the LAO surface and covered with a conductive protective coating to minimize surface charge accumulation on the insulating substrate. Using e-beam lithography, Hall bars with dimensions of 150 μm in width and 200 μm in length were patterned onto the LAO surface. After development, insulating AlO*_x_* films with a thickness of 70 nm were sputtered using AC sputtering to serve as a hard mask. After the lift-off process, 8-nm-thick LAO films were further grown in the PLD chamber, creating a conducting interface.

Preceding each LAO growth, the substrate was preannealed in situ at an oxygen pressure of 1 × 10^−4^ mbar and a temperature of 620°C for approximately 30 min. Amorphous LAO thin films were subsequently deposited from a single-crystalline target at the same temperature and pressure. The thickness of LAO films was controlled by counting the growth laser pulses. After growth, the LAO/KTO samples were cooled to room temperature under growth oxygen pressure. Finally, 10 nm of Ti and 50 nm of Au were sputtered onto the back of the KTO substrate using DC sputtering, serving as a back-gate electrode. All samples in this study shared the same device parameters.

### Transport measurements

Electrical contacts were established by connecting Au wires to the back-gate electrode, and direct ohmic contacts to the LAO/KTO(110) interface were achieved through ultrasonic bonding with Al wires. The applied current was parallel to the [1$\bar{1}$0] crystal axis of the KTO(110) substrate. To prevent potential Joule heating effect, all currents applied for resistance measurements were carefully chosen within the linear *I*-*V* region, as depicted in Fig. 1C (inset) and fig. S4B (inset), substantially below the critical current of the superconducting transition. To rule out the possibility that the quantum metal state originated from radiation thermalization, homemade 3.6-kHz resistor-capacitor filters with *R* = 1 kΩ and *C* = 47 nF were used to measure the *R*_S_(*T*) curves under various currents, as illustrated in Fig. 1C and fig. S4B. In addition, for the magnetoresistance measurements used in scaling analysis, a consistent sweeping direction from low to high magnetic fields with a sweep rate of typically 0.01 T/min was maintained to ensure a stable sample temperature. These measurements were conducted in an Oxford Triton dilution refrigerator.

## Supplementary Materials

### This PDF file includes:

Supplementary Text Figs. S1 to S18 Tables S1 and S2 References
