# Supplementary material for: Effectively tuning the quantum Griffiths phase by controllable quantum fluctuations
Source: Sci Adv. 2024 Nov 27;10(48):eadp1402. doi: 10.1126/sciadv.adp1402 (PMC11601250; doi:10.1126/sciadv.adp1402)
Supplement: Supplementary file 1 — Supplementary Text Figs. S1 to S18 Tables S1 and S2 References [file sciadv.adp1402_sm.pdf]

Science Advances
AAAS

Supplementary Materials for

Effectively tuning the quantum Griffiths phase by controllable quantum fluctuations

Beilin Wang *et al.*

Corresponding author: Changgan Zeng, cgzeng@ustc.edu.cn; Haiwen Liu, haiwen.liu@bnu.edu.cn

Sci. Adv. **10**, eadp1402 (2024)
DOI: 10.1126/sciadv.adp1402

This PDF file includes:

Supplementary Text
Figs. S1 to S18
Tables S1 and S2
References

Supplementary Text

S1. Definition of the upper critical magnetic field and the superconducting transition temperature

Theoretical investigations have revealed that superconducting fluctuations dominate the conductivity correction near the quantum critical point (*37-41*). The infinite-randomness quantum critical point (B_C^*) can be determined through the activated scaling law shown in Fig. 2F. To effectively analyze the influence of superconducting fluctuations on conductivity, it is necessary for the defined zero-temperature upper critical magnetic field $B_{C2}(T = 0)$ to approach the quantum critical point (detailed in Supplementary Text S5). To this end, we defined the upper critical magnetic field using the 90% normal-state resistance criterion. For consistency, the same criterion was applied to determine the transition temperature. Notably, this criterion has also been widely adopted in various two-dimensional (2D) superconductors (*14, 29, 60*).

S2. The validity of the finite-size analysis

The finite-size scaling (FSS) analysis we employed to determine effective critical exponents ($z\nu$) has been validated in numerous studies (*17, 22-29*). Furthermore, we have taken careful steps in both measurements and data processing, particularly concerning the determination of crossing points and critical exponents, to ensure the reliability of our scaling analysis.

Firstly, we meticulously controlled experimental conditions, maintaining a consistent sweeping direction from low to high magnetic fields at a small rate typically of 0.01 T/min. This ensured the robustness of the $R_S(B)$ curves across different temperatures.

Secondly, we rigorously adhered to the scaling analysis procedure. Specifically, the neighboring $R_S(B)$ curves are divided into groups with smaller temperature range (cf. Fig. S6), allowing us to precisely identify the crossing points (B_C, R_C). These crossing points, indicative of $\mathrm{d}R_S/\mathrm{d}T = 0$, were further crosschecked against the extreme points of the $R_S(T)$ curves at various magnetic fields (Figs. 2E and 3C). The resistivity was then analyzed by scaling analysis in each group. The high resolution of resistance (0.01 Ω) and magnetic field (0.1 mT) guaranteed the accuracy of effective critical exponents obtained in each group.

S3. Discussions on the quantum metal state and the quantum Griffiths phase

While we have identified the coexistence of quantum metal states and the quantum Griffiths phase (QGP), they are independent phenomena. This independence is consistent with the observation of QGP in various 2D superconductors that lack the quantum metal state (*22-29*). Previous studies have provided a phase diagram under a magnetic field to illustrate their coexistence relationship (*17, 36*).

The primary distinction between the two phenomena lies in their underlying mechanisms: the quantum metal state is induced by the combined effects of ohmic dissipation and phase fluctuations, whereas the QGP is induced by the combined effects of ohmic dissipation and amplitude fluctuations of the superconducting order parameter. In the quantum metal state (low magnetic field region), the order parameter has a relatively large value. In the presence of pronounced disorder, superconductivity can be locally disrupted to form inhomogeneous superconducting regions in the system, and weak Josephson links form between the superconducting puddles. Meanwhile, strong ohmic dissipation leads to phase damping, dominating the dynamic behavior and leading to saturation resistance at low temperatures (*36*). In contrast, in the case of QGP (high magnetic field region), locally ordered regions persist due to the quenched disorder effect, but the amplitude of order parameter is marginal around zero. The slow dynamics of these superconducting rare regions contribute to the divergent effective critical exponent.

For the LAO/KTO(110) interface adopted in our study, the quantum metal state consists of superconducting puddles (with a relatively large value of the order parameter) and their surrounding weak Josephson links. These links induce pronounced phase fluctuations and ohmic dissipation between neighboring superconducting puddles (*61*), and give rise to the quantum metal state with residual temperature-independent resistance in the ultralow temperature regime (Fig. 1B). Nevertheless, when increasing the magnetic field towards the quantum critical point, the magnetic field leads to the pair-breaking of Cooper pairs and the amplitude of order parameter approaches zero. Then, the combined effects of ohmic dissipation and quenched disorder effect result in the formation of large superconducting rare regions with

ultraslow dynamics, and the effective critical exponent ($z\nu$) is divergent when approaching zero temperature, namely the QGP (Fig. 2F). Thus, while both phenomena can coexist, they are driven by fundamentally different physical mechanisms and emerge in different parameter regimes.

S4. Discussions on the alternative mechanisms of the anomalous phase boundary

Several mechanisms can also give rise to the reentrant behavior of $R_S(T)$. The Werthamer-Helfand-Hohenberg scenario (*62*) is ruled out, as it fails to reproduce the anomalous phase boundary observed in our experiments (see Fig. S11). Additionally, the mechanism involving the competition between antiferromagnetism and superconductivity (*63*) is also dismissed, given the absence of any reported instances of antiferromagnetism in the LAO/KTO(110) interface. Lastly, reentrant behavior has also been documented in amorphous superconducting films, attributed to the competition between Josephson coupling and Coulomb interaction (*64*). However, this process typically results in negative magnetoresistance in the normal state at low temperatures, which differs from our results (Fig. 2C) and can thus be excluded.

S5. $R_S(T)$ fitting by superconducting fluctuation theory

Superconducting fluctuations in a 2D system consist of Cooper pairs with finite lifetimes that exist above the transition temperature but do not form a stable condensate. These fluctuations are typically described by four primary contributions. The first term is the Aslamazov-Larkin (AL) process, corresponding to the opening of a new channel for the charge transfer (*37*). The second term is the Maki-Thompson (MT) process (*65, 66*), which describes single-particle quantum interference at impurities in the presence of superconducting fluctuations, analogous to weak localization, except here two electrons form some specific fluctuation Cooper pair (*41*). The third term is the single-particle density of states (DOS) process due to their involvement in fluctuation pairings. Lastly, the diffusion coefficient renormalization (DCR) term. The corrections to conductivity from superconducting fluctuations in a dirty limit 2D superconductor have been elucidated in prior theoretical studies (*37, 38*). Recent full solutions for superconducting fluctuations in 2D superconductors without

spin-orbit coupling (SOC) have shown that the DCR term plays a key role in the fluctuations near the quantum critical point, which is related to the Cooperon propagator (*40*).

The KTO(110) heterointerfaces are known to accommodate a strong SOC (*51, 52*), which can modify the form of the Cooperon propagator by introducing the spin-triplet channel with a negative sign in addition to the spin-singlet channel with a positive sign (*50, 67*). In the following, we examine the effect of superconducting fluctuations with strong SOC on the conductivity. The total conductivity, as validated in previous studies of strong SOC systems (*26*), can be described as:

$$\sigma = \sigma_{\mathrm{N}} + \frac{e^2}{\pi^2\hbar}\left[\alpha I_\alpha(b,t) + \beta I_\beta(b,t)\right] + C\left[\exp\left(-\frac{\Delta}{k_B T}\right) - 1\right] \qquad (1)$$

with

$$I_\alpha(b,t) = \ln\frac{r}{b} - \frac{1}{2r} - \psi(r) \qquad (2)$$

and

$$I_\beta(b,t) = r\psi'(r) - \frac{1}{2r} - 1,$$

(3)

where $r = \frac{b}{3.562t}, t = T/T_{\mathrm{C}} \ll 1, b = \left[B - B_{\mathrm{C2}}(T)\right]/B_{\mathrm{C2}}(0) \ll 1, \psi(r)$ is the digamma function, and $B_{\mathrm{C2}}(T)$ is the upper critical magnetic field, given by the Werthamer-Helfand-Hohenberg theory (*62*). When the magnetic field increases much larger than the superconducting critical field, the contribution of the superconducting fluctuations becomes negligible, and the temperature dependence of the sheet resistance can be considered as the normal state resistance. Thus, $\sigma_{\mathrm{N}} = \sigma_0 + d \cdot \sigma_{\mathrm{1T}}$, where σ_{1T} is the sheet conductance of Sample #1 at 1 T. Δ is the activation energy of thermal excitation (the local superconducting pairing strength).

The transition temperature T_{C} of Sample #1 is approximately 0.76 K at V_{G} = -200 V. Therefore, we focused on fitting the data in the low-temperature region (< 0.6 K) to satisfy the condition $t = T/T_{\mathrm{c}} \ll 1$. The temperature-dependent sheet resistance $R_{\mathrm{S}}(T)$ curves at various magnetic fields ranging from 133 to 141 mT were successfully fitted using equation (1) (see Fig. 2B). The fitting parameters were summarized in Table S1, which agrees with the previous study on anomalous QGS (*26*).

The parameters α and β, estimated to be 0.08 and -0.016, respectively, differ from those reported in previous studies that did not consider the influence of SOC (*38*). The slight magnetic field dependence of α and β supports the reliability of our considerations (*26*). Regarding the DOS term, Δ remains stable, while the coefficient C decreases with increasing magnetic field due to a reduction in the fluctuation of Cooper pairs at higher magnetic fields. Overall, the theoretical phase boundary, determined from the minima of the fitted $R_S(T)$ curves ranging from 133 to 141 mT, is presented in Fig. 2E, aligning well with the experimental data.

Notably, the superconducting quantum fluctuations are mediated by electron-electron channels (the Cooperon channel), and in systems with large SOC the spin-triplet Cooperon channel plays a dominant role than the spin-singlet Cooperon channel, and leads to conductivity correction coefficients with opposite sign, similar to the case of weak anti-localization corrections in the surface state of 3D topological insulator (*68*). This conductivity correction from quantum fluctuations only needs the fluctuating spin-triplet Cooperon channels but not requires the formation of spin-triplet Cooper pairs into a coherent Bose-Einstein condensation state. As such, our findings do not conflict with the previous research on the superconductivity of the KTO-based interfaces (*69*). Additionally, we note a recent study on KTO-based interfaces that identified in-plane twofold symmetric oscillations in the upper critical magnetic field (*70*). This observation offers promising experimental evidence for the existence of spin-triplet component within the quantum fluctuation regime at the LAO/KTO interface.

S6. Direct activated scaling analysis

Based on previous investigations, the existence of QGS can be directly demonstrated by the direct activated scaling analysis (*25, 28, 29*). This analysis is expressed as follows: $R=\Phi\left\{\left[\left(B-B_{\mathrm{C}}^{*}\right)/B_{\mathrm{C}}^{*}\right]\left[\ln\left(T^{*}/T\right)\right]^{(1/v\psi)},u\left[\ln T^{*}/T\right]^{-(1/v\psi)-y}\right\}$, where v is the correlation length exponent, ψ is the tunneling critical exponent, T^{*} is the characteristic temperature of quantum fluctuation, B_{C}^{*} is the critical field at zero temperature, u is the leading irrelevant scaling variable and $y > 0$ is the associated irrelevant exponent. The irrelevant correction can influence

the phase boundary $B_C(T)$: $\left\{\left[B_c^* - B_c(T)\right]/B_c^*\right\} \propto u\left[\ln\left(T^*/T\right)\right]^{-(1/\nu\psi)-y}$. Thus, the parameters u, $\nu\psi$, T^* and B_C^* can be obtained by fitting the phase boundary, as shown in Fig. 3C. The corresponding fitting parameters are summarized in Table S2.

Taking the irrelevant scaling variable correction into account (*71*), the scaling function in the second argument (*25*) can be expanded as:

$$R(B,T) = \Phi_1\{[\left(B - B_c^*\right)/B_c^*\left]\right[\ln\left(T^*/T\right. + u\left[\ln\left(T^*/T\right)\right]^{-y}\Phi_2\left\{\left[\left(B - B_C^*\right)/B_C^*\right]\left[\ln\left(T^*/T\right)\right]^{(1/\nu\psi)}\right\}$$

Here Φ_1 and Φ_2 are arbitrary functions. We can define $x_1(B,T) = \delta[\ln(T^*/T)]^{(1/\nu\psi)}$, $x_2(T) = [\ln(T^*/T)]^{-y}$ and $\delta = |B - B_C^*|$. Thus, $R(B,T)$ can be rewritten as $R(B,T) = f(x_1) + x_2 \cdot g(x_1)$, and both x_1 and x_2 for given B and T can be calculated using fitting parameters in Table S2. First, we plot the $R(x_1)$ diagram from the $R_S(B)$ curves for various temperatures. After linearly interpolating $R(x_1)$ curves, we can obtain $R(T)|_{x_1=x_{10}} = f(x_{10}) + x_2(T) \cdot g(x_{10})$ for any fixed $x_1 = x_0$ (where x_0 is an arbitrary value of x_1). This suggests that $R(T)|_{x_1=x_{10}}$ and $x_2(T)$ are linearly related. The corrected resistance $\widetilde{R}(x_{10},T)$ for various temperatures can be defined as $R(x_{10},T) = R(T)|_{x_1=x_{10}} - k(x_{10}) \cdot x_2(T)$, where $k(x_{10})$ represents the slope of the $R(T)|_{x_1=x_{10}}$ vs $x_2(T)$ curve, obtained from linear fitting. Finally, $\widetilde{R}(x_{10},T)$ for various temperatures collapses onto the universal curves (cf. Fig. S16), providing direct evidence of QGS.

S7. Additional discussions on the anomalous phase boundary

Superconducting fluctuations with strong SOC can lead to the anomalous phase boundary. It is believed that the presence of relatively strong disorder further enhances superconducting fluctuations (*8, 26*). Generally, the degree of disorder can be assessed using the Ioffe-Regel parameter, denoted as $k_F l_{mfp}$ (*4*), where k_F is the fermi wave vector, and l_{mfp} is the mean free path. In the framework of the single-band model (*42, 43*), $l_{mfp} = h/(e^2 k_F R_N)$, where h is the Planck constant, e is the electron charge, and R_N is the normal-state sheet resistance.

Here, we provide a simple estimation of the Ioffe-Regel parameter for the current systems that have reported QGS based on the single-band model. In our work, the $k_F l_{mfp}$ for sample #1 at V_G = -200 V is estimated to be 7.6, a value comparable to the 9.6 obtained in the 3.5-

monolayer (ML) Pb film from the previous anomalous QGS study (*26*). In contrast, $k_F l_{mfp}$ values for systems showing normal QGS are estimated as follows: 29 for the 3-ML Ga film (*22*), 57.4 for the 1-ML $NbSe_2$ film (*24*), 32.2 for the LAO/$SrTiO_3$(110) interface (*23*), and 28.6 for the 4-ML $PbTe_2$ film (*28*). It is evident that systems exhibiting anomalous QGS demonstrate a higher degree of disorder compared to those displaying QGS, thereby suggesting the effect of superconducting fluctuations is more pronounced in these systems.

It is noteworthy that a prior study, adopting the same interface as ours, did not observe the anomalous phase boundary and reentrant behavior (*42*). The absence of these phenomena can be attributed to the less prominent effect of quantum fluctuations due to weaker disorder in their samples. The $k_F l_{mfp}$ of their samples is estimated at 21.5, three times higher than our samples at 7.6. This observation agrees with the comparative analysis of mobilities, with our mobility estimated at 43 $cm^2V^{-1}s^{-1}$ (Fig. S4C), significantly lower than theirs at 81 $cm^2V^{-1}s^{-1}$, collectively indicating a higher degree of disorder in our systems. Crucially, our study establishes the phase boundary based on the crossing points of $R_S(B)$ curves at neighboring temperatures, following the principle of FSS analysis. This phase boundary inherently differs from the temperature-dependent upper critical magnetic field defined in the prior study (*42*), which used a 50% normal-state resistance criterion.

In addition, it should be clarified that the disorder strength represented by the Ioffe-Regel parameter only reflects the disorder of normal state fermions, whereas the disorder strength in the context of QGS is indicative of the disorder in local Cooper pairing (which also undergoes renormalization under coarse-graining as the temperature approaches zero) (*28*). Nonetheless, the Ioffe-Regel parameter can still serve as a valuable reference value for evaluating the disorder strength in QGS.

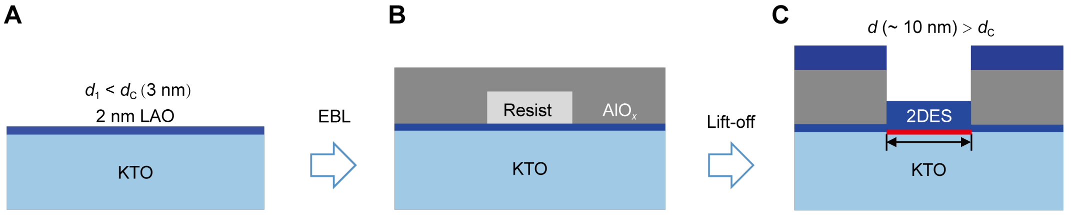

Fig. S1. Schematic of the fabrication process steps for the LAO/KTO(110) interface Hall device. (**A**) Deposition of approximately 2 nm of amorphous LAO on a KTO substrate, which is thinner than the critical thickness required for inducing the 2DES at the interface. (**B**) Growth of approximately 70 nm of AlO_x as a hard mask on a resist lift-off sample. (**C**) Deposition of approximately 8 nm of LAO. The red line indicates the area where the 2DES is formed at the LAO/KTO interface.

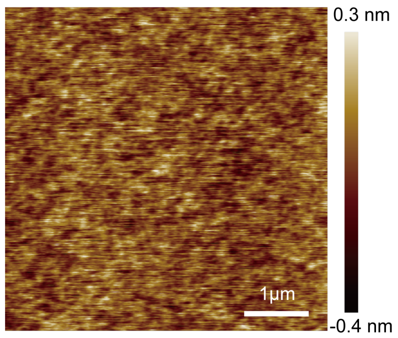

Fig. S2. Atomic force microscopy image of a 10-nm LAO/KTO sample. The surface is very smooth, with a root-mean-square roughness of about 0.1 nm over the whole 5 μm by 5 μm area.

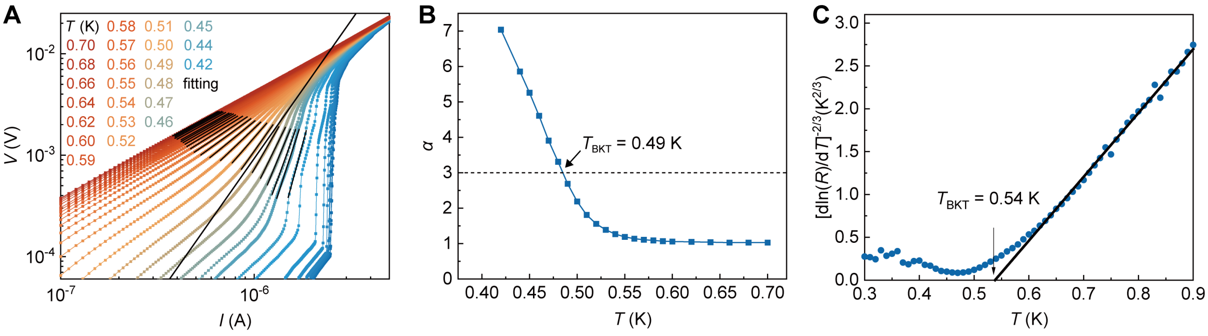

Fig. S3. Berezinskii-Kosterlitz-Thouless (BKT) transition of Sample #1. (**A**) *I*-*V* curves on a logarithmic scale were measured at different temperatures. Black lines show the $V{\sim}I^{\alpha}$ dependence, and the T_{BKT} can be estimated from the power-law exponent $\alpha = 3$ shown in (**B**).

(**C**) Sheet resistance can be expressed as $R_S(T) \propto \exp[-b(T/T_{BKT}-1)^{-1/2}]$ dependence when the temperature close to T_{BKT}, where b is the material parameter. Here, $R_S(T)$ is plotted on a $[d\ln(R_S)/dT]^{-2/3}$ scale. The solid line is the behavior expected for a BKT transition with T_{BKT} = 0.53 K.

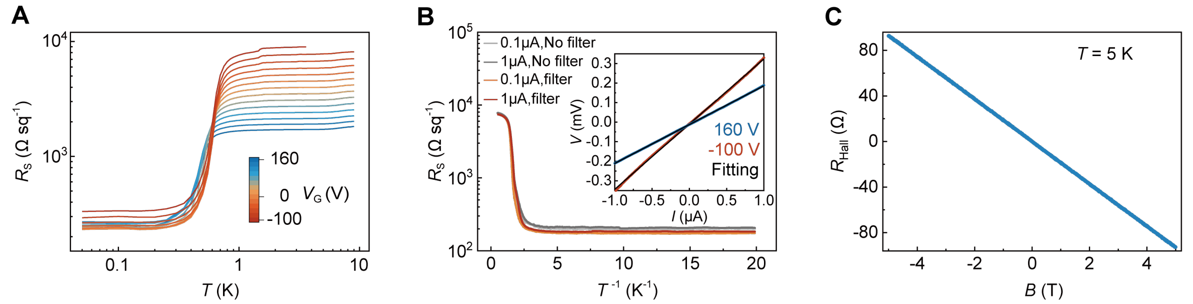

Fig. S4. Transport characterization of Sample #2. (**A**) Temperature dependence of the sheet resistance $R_S(T)$ at different gate voltages (V_G), showing the presence of residual resistance across the entire V_G range. (**B**) Arrhenius plot of the sheet resistance with and without resistor-capacitor filters for different currents at V_G = -100 V. Inset: typical linear I-V curves in the low current range (V_G = 160 V, -100 V). (**C**) Dependence of R_{Hall} on the magnetic field at T = 5 K.

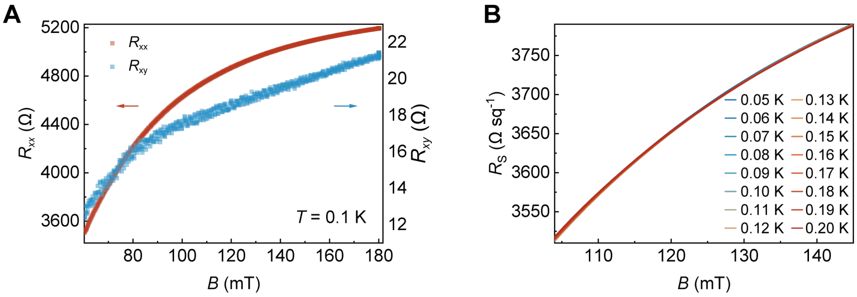

Fig. S5. Magnetic field dependence of the longitudinal resistance and Hall resistance of Sample #1. (**A**) A remarkable Hall resistance (R_{xy}) near the crossing region of Fig. 2C at V_G = -200 V indicates a pair-breaking-induced quantum phase transition. (**B**) Sheet resistance as a function of the perpendicular magnetic field at various temperatures.

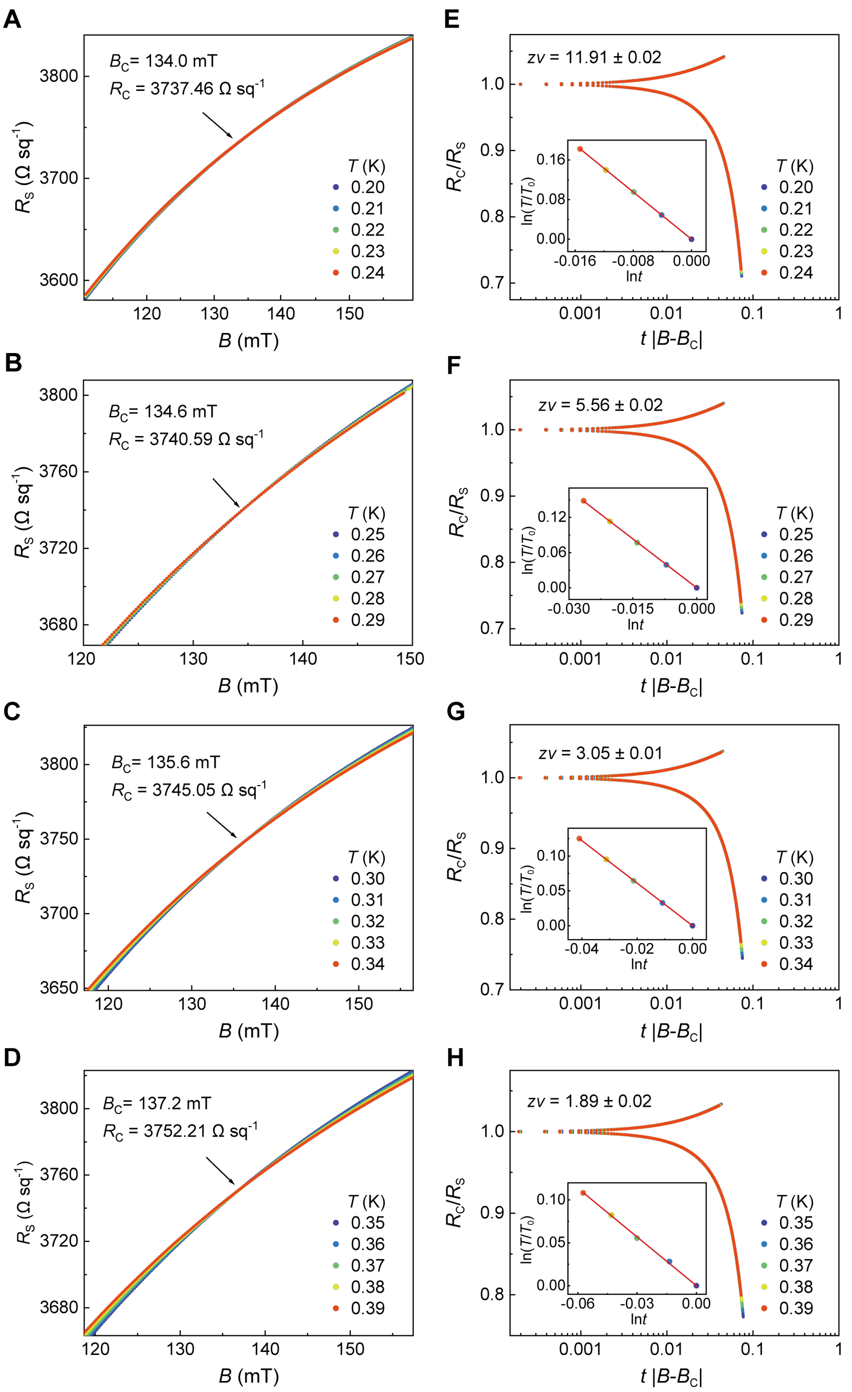

Fig. S6. FSS analysis for Sample #1 at temperatures ranging from 0.20 to 0.39 K. (**A** to **D**) Sheet resistance as a function of the perpendicular magnetic field at various temperature ranges. (**E** to **H**) Corresponding normalized resistance as a function of the scaling variable $t\,|B - B_C|$, with $t = (T/T_0)^{-1/z\nu}$. Inset: linear fitting between ln(T/T_0) and ln(t) gives the critical exponent. B_c is determined by the crossing points of the $R_S(B)$ curves, and T_0 is the lowest temperature in each set of $R_S(B)$ curves.

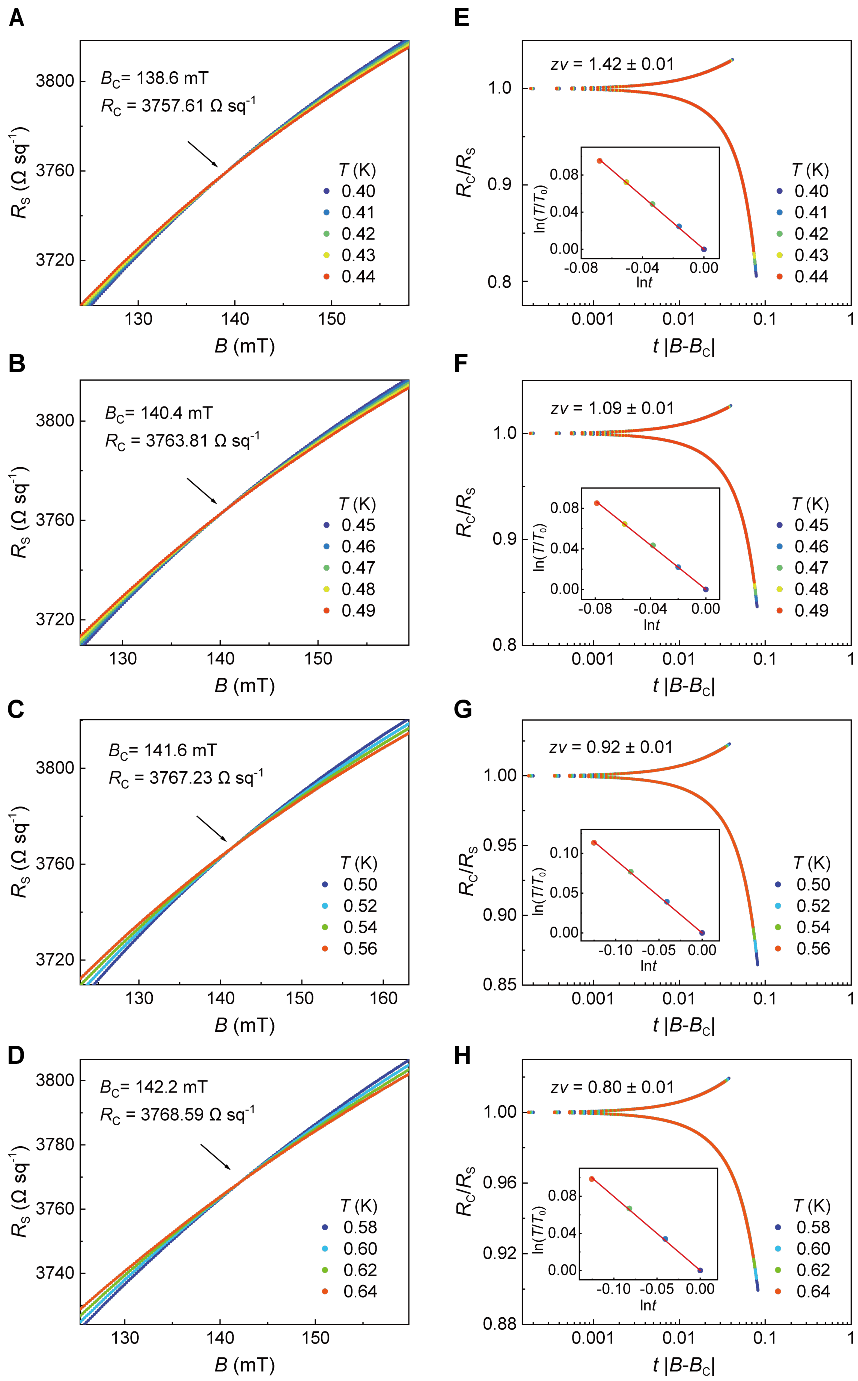

Fig. S7. FSS analysis for Sample #1 at temperatures ranging from 0.40 to 0.64 K. (**A** to **D**) Sheet resistance as a function of the perpendicular magnetic field at various temperature ranges. (**E** to **H**) Corresponding normalized resistance as a function of the scaling variable $t\,|B - B_C|$, with $t = \left(T / T_0\right)^{-1/z\nu}$. Inset: linear fitting between ln(T/T_0) and ln(t) gives the critical exponent. B_c is determined by the crossing points of the $R_S(B)$ curves, and T_0 is the lowest temperature in each set of $R_S(B)$ curves.

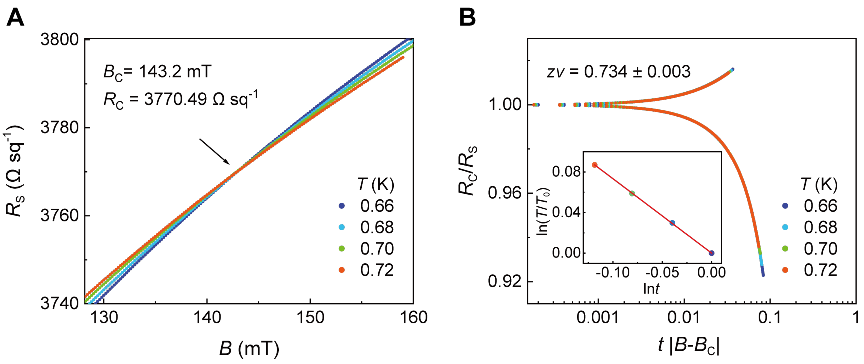

Fig. S8. FSS analysis for Sample #1 at temperatures ranging from 0.66 to 0.72 K. (**A**) Sheet resistance as a function of the perpendicular magnetic field at various temperature ranges. (**B**) Corresponding normalized resistance as a function of the scaling variable $t\,|B-B_C|$, with $t=\left(T/T_0\right)^{-1/z\nu}$. Inset: linear fitting between ln(T/T_0) and ln(t) gives the critical exponent. B_c is determined by the crossing points of the $R_S(B)$ curves, and T_0 is the lowest temperature in each set of $R_S(B)$ curves.

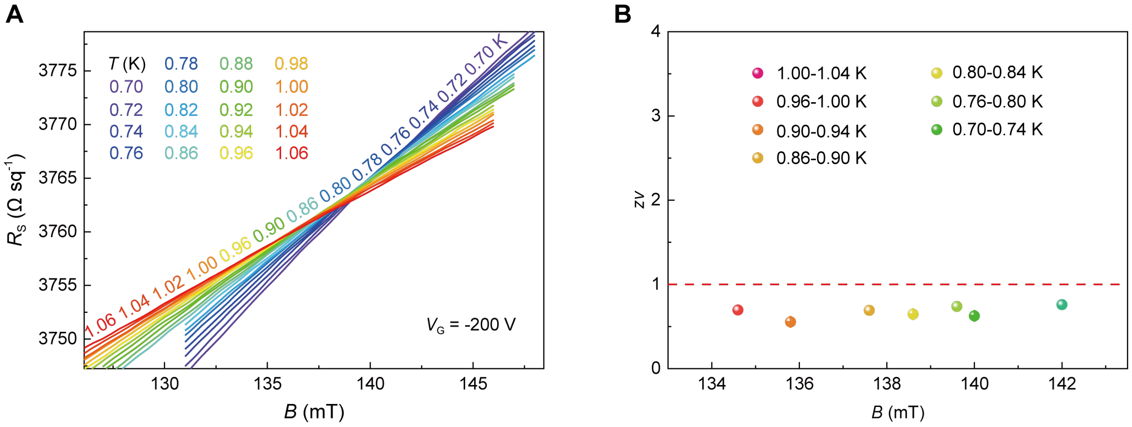

Fig. S9. Magnetoresistance and the effective critical exponent at relatively high temperatures for Sample #1. (**A**) $R_S(B)$ curves at temperatures ranging from 0.70 to 1.06 K in 0.02 K steps, exhibiting a pronounced transition region rather than a critical point. (**B**) Critical exponents from 0.70 to 1.04 K remain small (<1) and relatively stable, as the temperature is close to T_{C0} and far from the quantum critical regime.

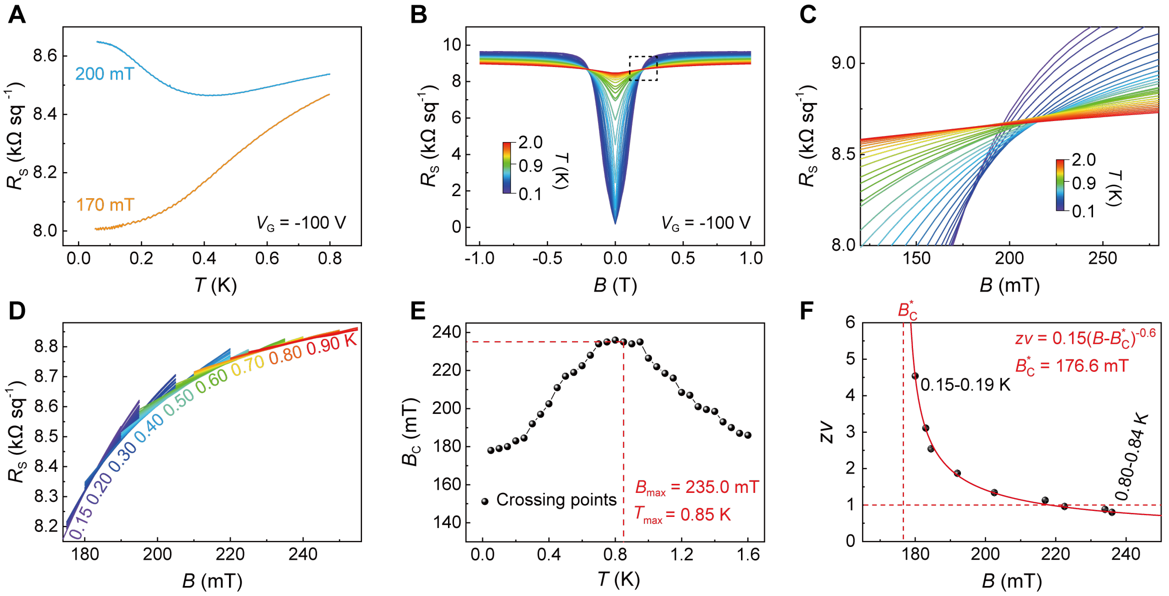

Fig. S10. Anomalous QGS for Sample #2 under perpendicular magnetic fields. (**A**) $R_S(T)$ curves measured at different magnetic fields, revealing clear reentrant behavior. (**B**) $R_S(T)$ curves measured with temperatures ranging from 0.1 to 1.0 K in 0.05 K steps, and from 1.0 to 2.0 K in 0.1 K steps. (**C**) A zoomed-in view of the crossing region in (**B**). (**D**) $R_S(B)$ curves measured for temperatures ranging from 0.15 to 0.90 K in 0.01 K steps. (**E**) Crossing points $B_C(T)$ obtained from neighboring $R_S(B)$ curves (black points). (**F**) Critical exponent zv obtained from FSS analysis as a function of the magnetic field follows the activated scaling law (red solid line). Scaling analysis reveals anomalous QGS behavior. Error bars represent the width of the zv value are acquired during the scaling analysis.

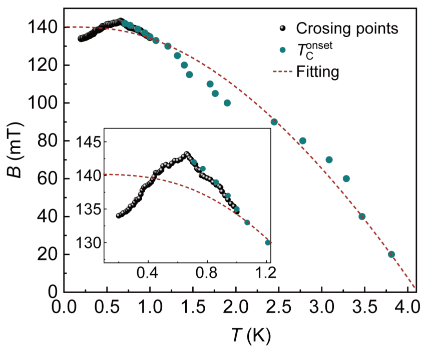

Fig. S11. Phase boundary for Sample #1 fitted by the Werthamer-Helfand-Hohenberg model. The red dashed line shows the fitting based on the Werthamer-Helfand-Hohenberg model, which fails to reproduce the anomalous phase boundary (inset) observed in our study.

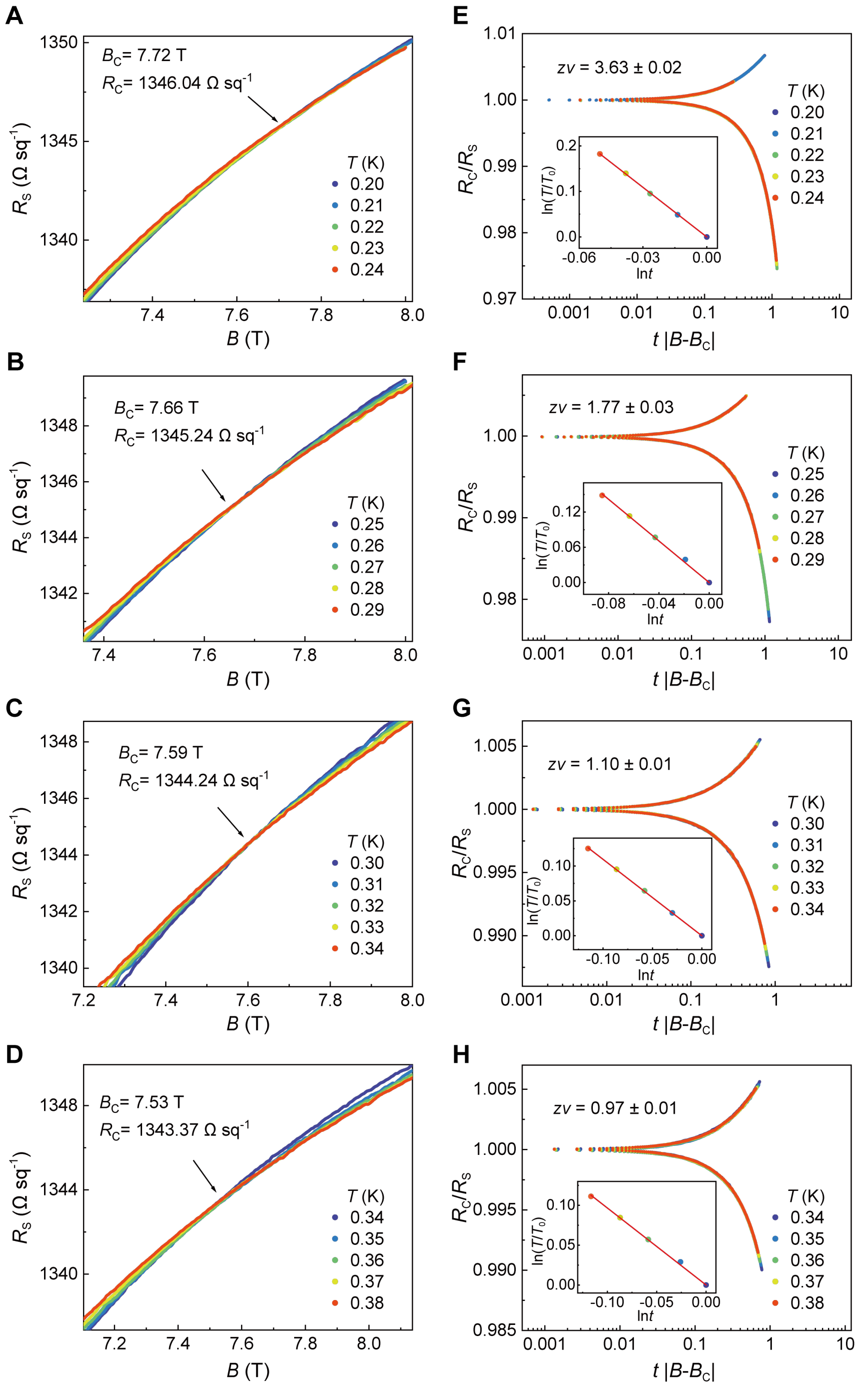

Fig. S12. FSS analysis for Sample #1 at temperatures ranging from 0.20 to 0.38 K. (**A** to **D**) Sheet resistance as a function of the perpendicular magnetic field at various temperature ranges. (**E** to **H**) Corresponding normalized resistance as a function of the scaling variable $t\,|B-B_C|$, with $t=\left(T/T_0\right)^{-1/z\nu}$. Inset: linear fitting between ln(T/T_0) and ln(t) gives the critical exponent. B_c is determined by the crossing points of the $R_S(B)$ curves, and T_0 is the lowest temperature in each set of $R_S(B)$ curves.

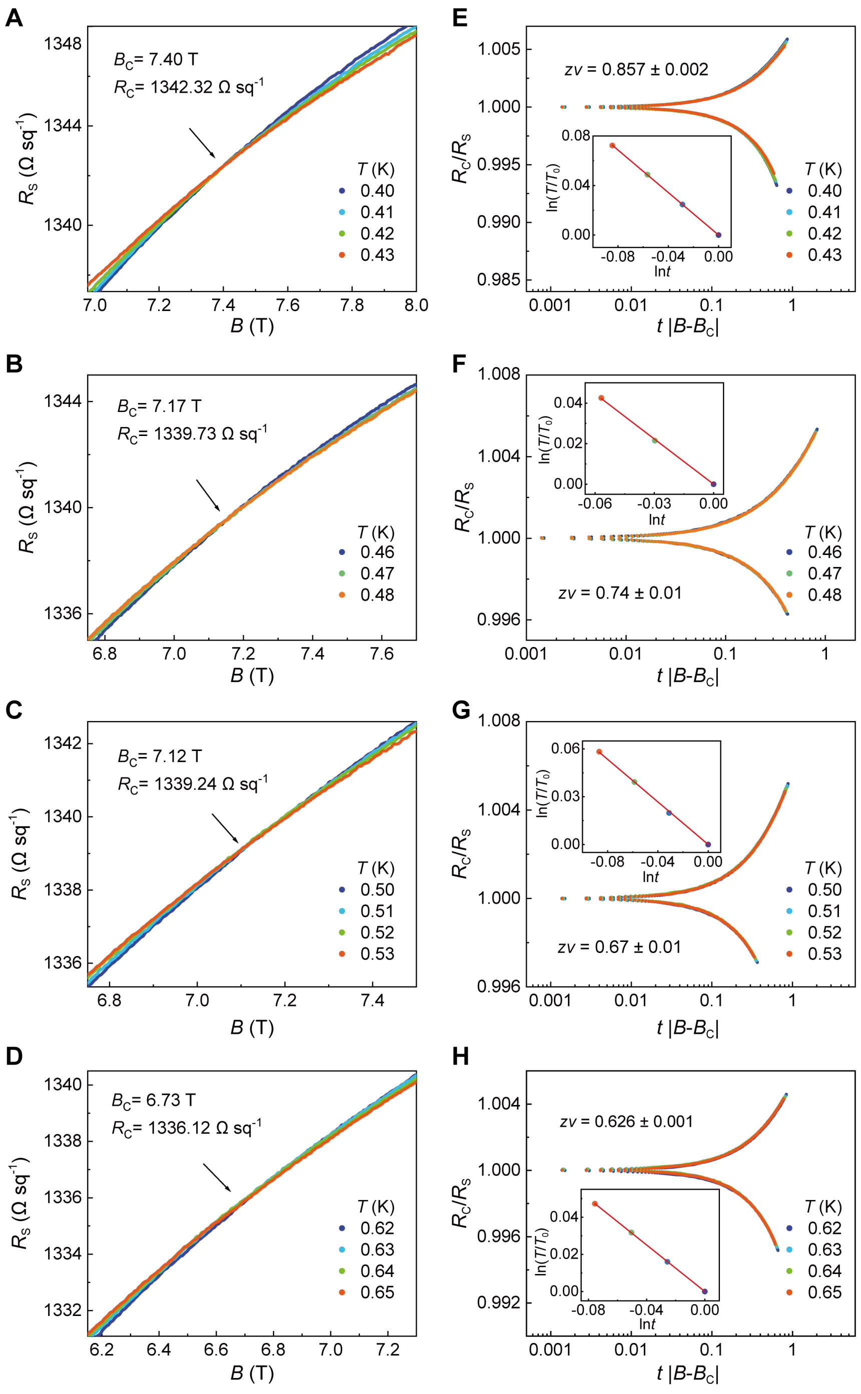

Fig. S13. FSS analysis for Sample #1 at temperatures ranging from 0.40 to 0.65 K. (**A** to **D**) Sheet resistance as a function of the perpendicular magnetic field at various temperature ranges. (**E** to **H**) Corresponding normalized resistance as a function of the scaling variable $t\,|\,B - B_C\,|$, with $t = \left(T / T_0\right)^{-1/z\nu}$. Inset: linear fitting between ln(T/T_0) and ln(t) gives the critical exponent. B_c is determined by the crossing points of the $R_S(B)$ curves, and T_0 is the lowest temperature in each set of $R_S(B)$ curves.

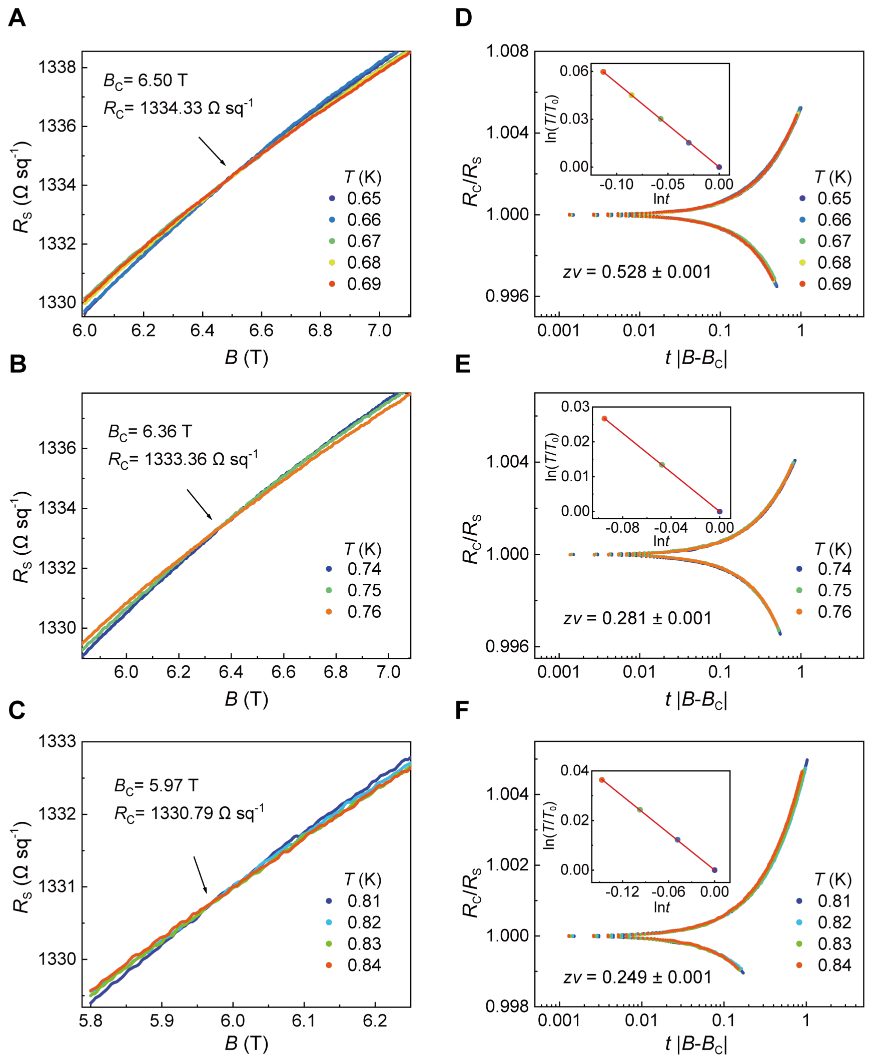

Fig. S14. FSS analysis for Sample #1 at temperatures ranging from 0.65 to 0.84 K. (**A** to **C**) Sheet resistance as a function of the perpendicular magnetic field at various temperature ranges. (**D** to **F**) Corresponding normalized resistance as a function of the scaling variable $t\,|\,B - B_C\,|$, with $t = \left(T/T_0\right)^{-1/z\nu}$. Inset: linear fitting between ln(T/T_0) and ln(t) gives the critical exponent. B_c is determined by the crossing points of the $R_S(B)$ curves, and T_0 is the lowest temperature in each set of $R_S(B)$ curves.

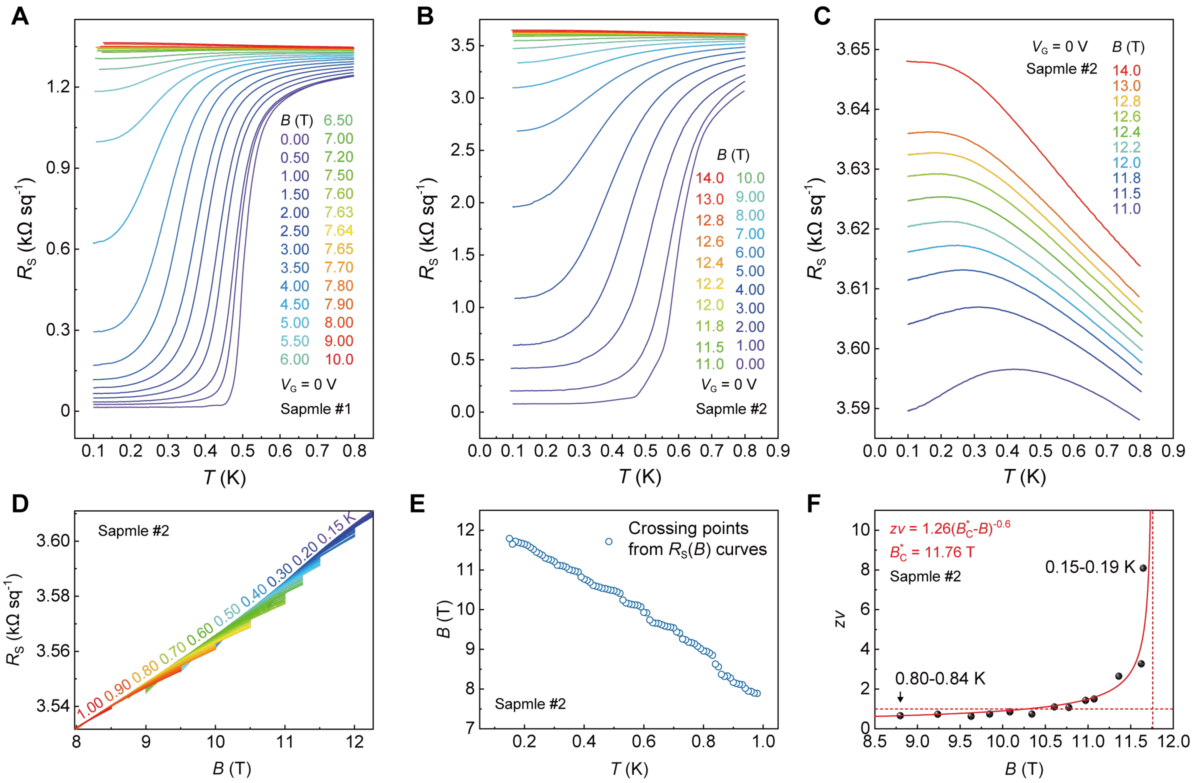

Fig. S15. Transport properties measured under parallel magnetic fields. (**A**), (**B**) Sheet resistance as a function of temperature at various parallel magnetic fields for Sample #1 and Sample #2, respectively. (**C**) $R_S(T)$ curves extracted from (B) show no sign of reentrant behavior. (**D**) $R_S(B)$ curves systematically measured at the temperatures ranging from 0.15 to 1.00 K. (**E**) Crossing points B_C(T) obtained from neighboring $R_S(B)$ curves in (**D**). (**F**) Critical exponent zν obtained from FSS analysis as a function of the magnetic field follows the activated scaling law (red solid line).

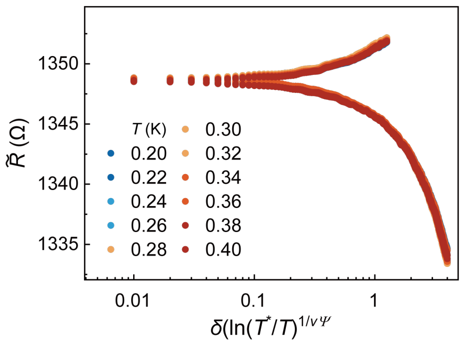

Fig. S16. Direct activated scaling analysis for Sample #1 from 0.20 to 0.40 K under parallel magnetic fields. $\widetilde{R}$ represents the corrected sheet resistance considering the irrelevant correction and $\delta = \left|B - B_{\mathrm{C}}^{*}\right|$. The details are described in Supplementary Text S6 and the parameters are listed in Table S2.

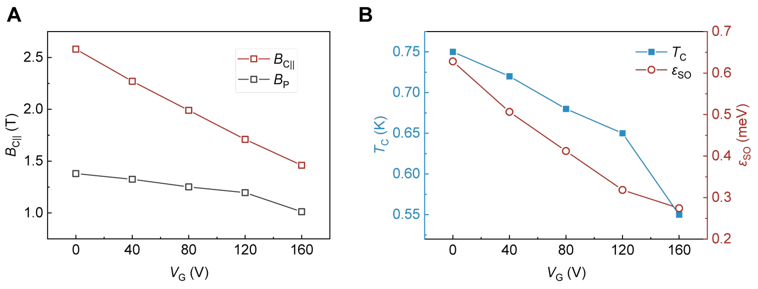

Fig. S17. Determination the SOC strength based on the spin-orbit scattering model. (**A**) Parallel upper critical field $B_{\mathrm{C}\|}$ and Pauli limiting field B_{P} at 0.15 K as a function of V_{G}. (**B**) T_{C} and SOC energy $\varepsilon_{\mathrm{SO}}$ as a function of V_{G}.

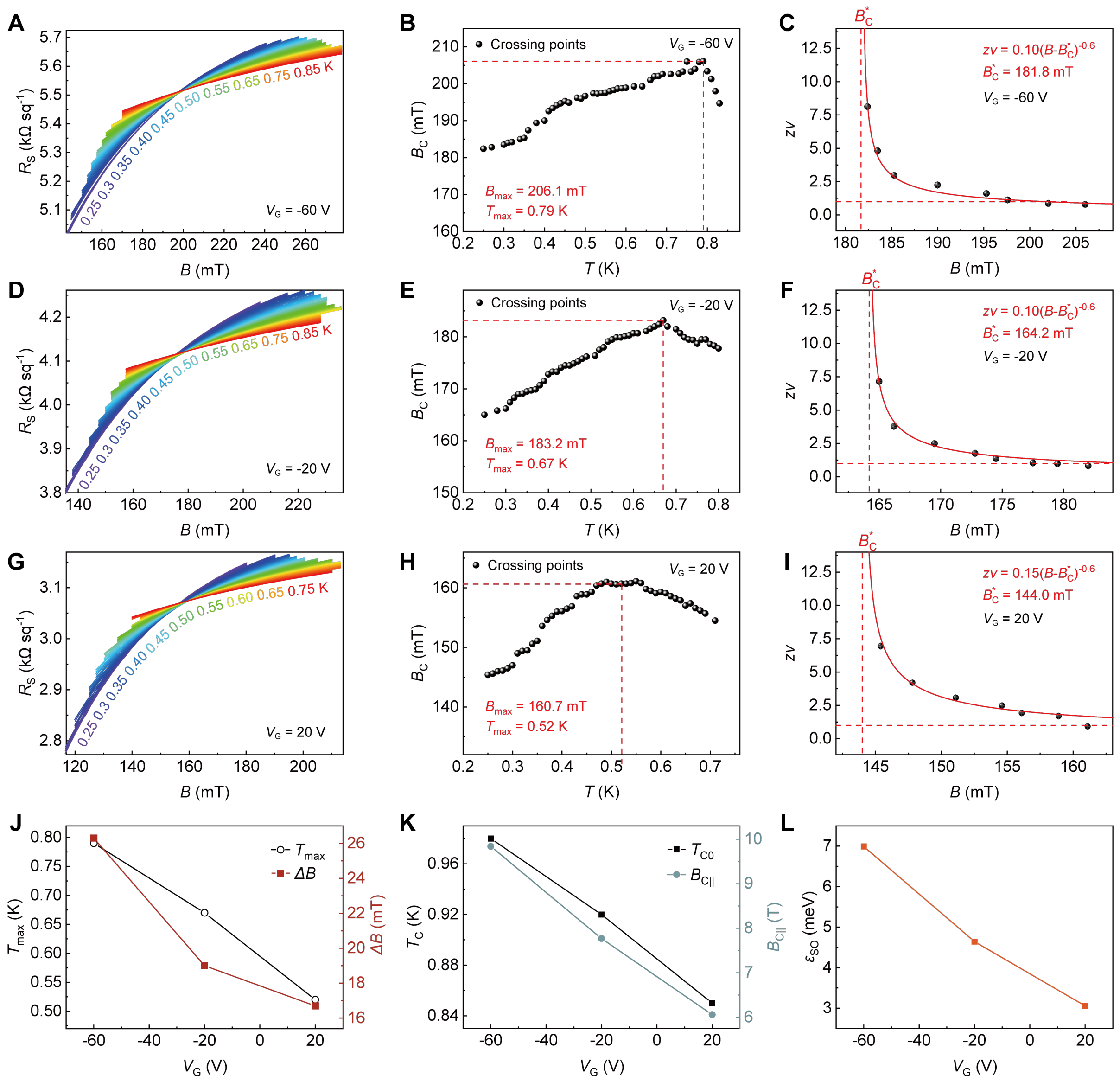

Fig. S18. V_G modulation of the anomalous phase boundary and spin-orbit couping energy. (**A**, **D**, and **G**) $R_S(B)$ curves measured for different temperatures in 0.01 K steps for V_G = -60, -20, 0 V, respectively. (**B**, **E**, and **H**) Crossing points $B_C(T)$ obtained from neighboring $R_S(B)$ curves for V_G = -60, -20, 0 V, respectively. (**C**, **F**, and **I**) Critical exponent $z\nu$ as a function of the perpendicular magnetic field for V_G = -60, -20, 0 V, respectively. Red solid lines show the activated scaling law $z\nu \propto \left|B - B_C^*\right|^{-0.6}$. (**J**) T_{max} and ΔB as a function of V_G, where T_{max} and $\Delta B = B_{max} - B_c^*$ represent the temperature and magnetic field ranges of the anomalous phase boundary, respectively. (**K**) T_C and parallel upper critical field $B_{C\parallel}$ (measured at 0.15 K) as a function of V_G. (**L**) SOC energy ε_{SO} as a function of V_G. All measurements were performed in Sample #4.

Table S1. Fitting parameters of equation (1) at perpendicular magnetic fields ranging from 133 to 141 mT for Sample #1.

B (mT)	α	β	C (Ω^{-1})	Δ/k_B (K)	σ_0 (Ω^{-1})	d
133	0.080	-0.024	2.94E-06	1.620	1.95E-04	0.045
135	0.080	-0.017	2.46E-06	1.615	1.79E-04	0.120
137	0.080	-0.015	2.15E-06	1.619	1.59E-04	0.225
139	0.085	-0.016	2.08E-06	1.619	1.35E-04	0.350
141	0.081	-0.016	1.46E-06	1.619	1.15E-04	0.450

Table S2. Fitting parameters of the activated scaling analysis the under parallel field for Sample #1.

B_C (T)	T^* (K)	$\nu\psi$	y	u
7.77	3.87	0.6	1.34	1.01

REFERENCES AND NOTES

1. S. L. Sondhi, S. M. Girvin, J. P. Carini, D. Shahar, Continuous quantum phase transitions. *Rev. Mod. Phys.* **69**, 315–333 (1997).

2. A. M. Goldman, Superconductor-insulator transitions. *Int. J. Mod. Phys. B* **24**, 4081–4101 (2010).

3. A. T. Bollinger, G. Dubuis, J. Yoon, D. Pavuna, J. Misewich, I. Božović, Superconductor-insulator transition in $La_{2-x}Sr_xCuO_4$ at the pair quantum resistance. *Nature* **472**, 458–460 (2011).

4. Y. Saito, Y. Kasahara, J. Ye, Y. Iwasa, T. Nojima, Metallic ground state in an ion-gated two-dimensional superconductor. *Science* **350**, 409–413 (2015).

5. Y. Cao, V. Fatemi, S. Fang, K. Watanabe, T. Taniguchi, E. Kaxiras, P. Jarillo-Herrero, Unconventional superconductivity in magic-angle graphene superlattices. *Nature* **556**, 43–50 (2018).

6. Z. Chen, Y. Liu, H. Zhang, Z. R. Liu, H. Tian, Y. Q. Sun, M. Zhang, Y. Zhou, J. R. Sun, Y. W. Xie, Electric field control of superconductivity at the $LaAlO_3/KTaO_3$(111) interface. *Science* **372**, 721–724 (2021).

7. Y. Dubi, Y. Meir, Y. Avishai, Nature of the superconductor-insulator transition in disordered superconductors. *Nature* **449**, 876–880 (2007).

8. B. Sacépé, M. Feigel’man, T. M. Klapwijk, Quantum breakdown of superconductivity in low-dimensional materials. *Nat. Phys.* **16**, 734–746 (2020).

9. H. M. Jaeger, D. B. Haviland, B. G. Orr, A. M. Goldman, Onset of superconductivity in ultrathin granular metal films. *Phys. Rev. B* **40**, 182–196 (1989).

10. A. Yazdani, A. Kapitulnik, Superconducting-insulating transition in two-dimensional *a*-MoGe thin films. *Phys. Rev. Lett.* **74**, 3037–3040 (1995).

11. D. Ephron, A. Yazdani, A. Kapitulnik, M. R. Beasley, Observation of quantum dissipation in the vortex state of a highly disordered superconducting thin film. *Phys. Rev. Lett.* **76**, 1529–1532 (1996).

12. N. Mason, A. Kapitulnik, Dissipation effects on the superconductor-insulator transition in 2D superconductors. *Phys. Rev. Lett.* **82**, 5341–5344 (1999).

13. C. Christiansen, L. M. Hernandez, A. M. Goldman, Evidence of collective charge behavior in the insulating state of ultrathin films of superconducting metals. *Phys. Rev. Lett.* **88**, 037004 (2002).

14. A. W. Tsen, B. Hunt, Y. D. Kim, Z. J. Yuan, S. Jia, R. J. Cava, J. Hone, P. Kim, C. R. Dean, A. N. Pasupathy, Nature of the quantum metal in a two-dimensional crystalline superconductor. *Nat. Phys.* **12**, 208–212 (2015).

15. N. P. Breznay, A. Kapitulnik, Particle-hole symmetry reveals failed superconductivity in the metallic phase of two-dimensional superconducting films. *Sci. Adv.* **3**, e1700612 (2017).

16. Z. Chen, A. G. Swartz, H. Yoon, H. Inoue, T. A. Merz, D. Lu, Y. Xie, H. Yuan, Y. Hikita, S. Raghu, H. Y. Hwang, Carrier density and disorder tuned superconductor-metal transition in a two-dimensional electron system. *Nat. Commun.* **9**, 4008 (2018).

17. Y. Saito, T. Nojima, Y. Iwasa, Quantum phase transitions in highly crystalline two-dimensional superconductors. *Nat. Commun.* **9**, 778 (2018).

18. L. Li, C. Chen, K. Watanabe, T. Taniguchi, Y. Zheng, Z. Xu, V. M. Pereira, K. P. Loh, A. H. Castro Neto, Anomalous quantum metal in a 2D crystalline superconductor with electronic phase nonuniformity. *Nano Lett.* **19**, 4126–4133 (2019).

19. C. Yang, Y. Liu, Y. Wang, L. Feng, Q. M. He, J. Sun, Y. Tang, C. C. Wu, J. Xiong, W. L. Zhang, X. Lin, H. Yao, H. W. Liu, G. Fernandes, J. Xu, J. M. Valles, J. Wang, Y. R. Li, Intermediate bosonic metallic state in the superconductor-insulator transition. *Science* **366**, 1505–1509 (2019).

20. A. Kapitulnik, S. A. Kivelson, B. Spivak, *Colloquium*: Anomalous metals: Failed superconductors. *Rev. Mod. Phys.* **91**, 011002 (2019).

21. Z. Chen, Y. Wang, A. G. Swartz, H. Yoon, Y. Hikita, S. Raghu, H. Y. Hwang, Universal behavior of the bosonic metallic ground state in a two-dimensional superconductor. *npj Quantum Mater.* **6**, 15 (2021).

22. Y. Xing, H. M. Zhang, H. L. Fu, H. W. Liu, Y. Sun, J. P. Peng, F. Wang, X. Lin, X. C. Ma, Q. K. Xue, J. Wang, X. C. Xie, Quantum Griffiths singularity of superconductor-metal transition in Ga thin films. *Science* **350**, 542–545 (2015).

23. S. C. Shen, Y. Xing, P. J. Wang, H. W. Liu, H. L. Fu, Y. W. Zhang, L. He, X. C. Xie, X. Lin, J. C. Nie, J. Wang, Observation of quantum Griffiths singularity and ferromagnetism at the superconducting $LaAlO_3/SrTiO_3(110)$ interface. *Phys. Rev. B* **94**, 144517 (2016).

24. Y. Xing, K. Zhao, P. Shan, F. Zheng, Y. Zhang, H. Fu, Y. Liu, M. Tian, C. Xi, H. Liu, J. Feng, X. Lin, S. Ji, X. Chen, Q. K. Xue, J. Wang, Ising superconductivity and quantum phase transition in macro-size monolayer $NbSe_2$. *Nano Lett.* **17**, 6802–6807 (2017).

25. N. A. Lewellyn, I. M. Percher, J. J. Nelson, J. Garcia-Barriocanal, I. Volotsenko, A. Frydman, T. Vojta, A. M. Goldman, Infinite-randomness fixed point of the quantum superconductor-metal transitions in amorphous thin films. *Phys. Rev. B* **99**, 054515 (2019).

26. Y. Liu, Z. Wang, P. Shan, Y. Tang, C. Liu, C. Chen, Y. Xing, Q. Wang, H. Liu, X. Lin, X. C. Xie, J. Wang, Anomalous quantum Griffiths singularity in ultrathin crystalline lead films. *Nat. Commun.* **10**, 3633 (2019).

27. Y. Ma, J. Niu, W. Xing, Y. Yao, R. Cai, J. Sun, X. C. Xie, X. Lin, W. Han, Superconductor-metal quantum transition at the $EuO/KTaO_3$ interface. *Chin. Phys. Lett.* **37**, 117401 (2020).

28. Y. Liu, S. Qi, J. Fang, J. Sun, C. Liu, Y. Liu, J. Qi, Y. Xing, H. Liu, X. Lin, L. Wang, Q. K. Xue, X. C. Xie, J. Wang, Observation of in-plane quantum griffiths singularity in two-dimensional crystalline superconductors. *Phys. Rev. Lett.* **127**, 137001 (2021).

29. C. Huang, E. Z. Zhang, Y. Zhang, J. L. Zhang, F. X. Xiu, H. W. Liu, X. Y. Xie, L. F. Ai, Y. K. Yang, M. H. Zhao, J. J. Qi, L. Li, S. S. Liu, Z. H. Li, R. Z. Zhan, Y. Q. Bie, X. F. Kou, S. Z. Deng, X. C. Xie, Observation of thickness-tuned universality class in superconducting β-W thin films. *Sci. Bull.* **66**, 1830–1838 (2021).

30. T. Vojta, Rare region effects at classical, quantum and nonequilibrium phase transitions. *J. Phys. A Math. Gen.* **39**, R143–R205 (2006).

31. T. Vojta, C. Kotabage, J. A. Hoyos, Infinite-randomness quantum critical points induced by dissipation. *Phys. Rev. B* **79**, 024401 (2009).

32. D. S. Fisher, Critical behavior of random transverse-field Ising spin chains. *Phys. Rev. B* **51**, 6411–6461 (1995).

33. T. Vojta, Disorder-induced rounding of certain quantum phase transitions. *Phys. Rev. Lett.* **90**, 107202 (2003).

34. T. Vojta, Quantum Griffiths effects and smeared phase transitions in metals: Theory and experiment. *J. Low Temp. Phys.* **161**, 299–323 (2010).

35. Y. Saito, T. Nojima, Y. Iwasa, Highly crystalline 2D superconductors. *Nat. Rev. Mater.* **2**, 16094 (2017).

36. Z. Wang, Y. Liu, C. Ji, J. Wang, Quantum phase transitions in two-dimensional superconductors: A review on recent experimental progress. *Rep. Prog. Phys.* **87**, 014502 (2024).

37. L. G. Aslamazov, A. I. Larkin, Effect of fluctuations on the properties of a superconductor above the critical temperature. *Fiz. Tverd. Tela (Leningr.)* **10**, 1104 (1968).

38. V. M. Galitski, A. I. Larkin, Superconducting fluctuations at low temperature. *Phys. Rev. B* **63**, 174506 (2001).

39. A. Larkin, A. Varlamov, *Theory of Fluctuations in Superconductors* (Clarendon Press, 2005).

40. A. Glatz, A. A. Varlamov, V. M. Vinokur, Fluctuation spectroscopy of disordered two-dimensional superconductors. *Phys. Rev. B* **84**, 104510 (2011).

41. A. A. Varlamov, A. Galda, A. Glatz, Fluctuation spectroscopy: From Rayleigh-Jeans waves to Abrikosov vortex clusters. *Rev. Mod. Phys.* **90**, 015009 (2018).

42. Z. Chen, Z. R. Liu, Y. Q. Sun, X. X. Chen, Y. Liu, H. Zhang, H. K. Li, M. Zhang, S. Y. Hong, T. S. Ren, C. Zhang, H. Tian, Y. Zhou, J. R. Sun, Y. W. Xie, Two-dimensional superconductivity at the $LaAlO_3/KTaO_3$(110) heterointerface. *Phys. Rev. Lett.* **126**, 026802 (2021).

43. C. J. Liu, X. Yan, D. F. Jin, Y. Ma, H. W. Hsiao, Y. L. Lin, T. M. Bretz-Sullivan, X. J. Zhou, J. Pearson, B. Fisher, J. S. Jiang, W. Han, J. M. Zuo, J. G. Wen, D. D. Fong, J. R. Sun, H. Zhou, A. Bhattacharya, Two-dimensional superconductivity and anisotropic transport at $KTaO_3$(111) interfaces. *Science* **371**, 716–721 (2021).

44. Y. Sun, Y. Liu, W. Pan, Y. Xie, Effects of growth temperature, oxygen pressure, laser fluence and postannealing on transport properties of superconducting $LaAlO_3/KTaO_3$(111) interfaces. *J. Phys. Condens. Matter* **34**, 444004 (2022).

45. J. Biscaras, N. Bergeal, S. Hurand, C. Feuillet-Palma, A. Rastogi, R. C. Budhani, M. Grilli, S. Caprara, J. Lesueur, Multiple quantum criticality in a two-dimensional superconductor. *Nat. Mater.* **12**, 542–548 (2013).

46. T. Vojta, A. Farquhar, J. Mast, Infinite-randomness critical point in the two-dimensional disordered contact process. *Phys. Rev. E* **79**, 011111 (2009).

47. I. A. Kovács, F. Iglói, Renormalization group study of the two-dimensional random transverse-field Ising model. *Phys. Rev. B* **82**, 054437 (2010).

48. J. A. Hoyos, T. Vojta, Theory of smeared quantum phase transitions. *Phys. Rev. Lett.* **100**, 240601 (2008).

49. V. M. Galitski, A. I. Larkin, Disorder and quantum fluctuations in superconducting films in strong magnetic fields. *Phys. Rev. Lett.* **87**, 087001 (2001).

50. L. P. Gor'kov, E. I. Rashba, Superconducting 2D system with lifted spin degeneracy: Mixed singlet-triplet state. *Phys. Rev. Lett.* **87**, 037004 (2001).

51. X. Y. Hua, F. B. Meng, Z. Y. Huang, Z. H. Li, S. Wang, B. H. Ge, Z. J. Xiang, X. H. Chen, Tunable two-dimensional superconductivity and spin-orbit coupling at the EuO/$KTaO_3$(110) interface. *npj Quantum Mater.* **7**, 97 (2022).

52. Y. Gan, F. Yang, L. Kong, X. Chen, H. Xu, J. Zhao, G. Li, Y. Zhao, L. Yan, Z. Zhong, Y. Chen, H. Ding, Light-induced giant rashba spin–orbit coupling at superconducting $KTaO_3$(110) heterointerfaces. *Adv. Mater.* **35**, e2300582 (2023).

53. A. M. Clogston, Upper limit for the critical field in hard superconductors. *Phys. Rev. Lett.* **9**, 266–267 (1962).

54. B. S. Chandrasekhar, A note on the maximum critical field of high-field superconductors. *Appl. Phys. Lett.* **1**, 7–8 (1962).

55. R. A. Klemm, A. Luther, M. R. Beasley, Theory of the upper critical field in layered superconductors. *Phys. Rev. B* **12**, 877–891 (1975).

56. P. K. Rout, E. Maniv, Y. Dagan, Link between the superconducting dome and spin-orbit interaction in the (111) $LaAlO_3$/$SrTiO_3$ interface. *Phys. Rev. Lett.* **119**, 237002 (2017).

57. A. K. Singh, T.-C. Wu, M.-Y. Song, M.-C. Chen, C.-S. Li, S. K. Yip, W.-L. Lee, Determination of spin-orbit scattering lifetime at the interface of $LaAlO_3/SrTiO_3$ from the superconducting upper critical fields. *Phys. Rev. Res.* **2**, 013311 (2020).

58. S.-g. Cheng, Y. Xing, J. Wang, Q.-f. Sun, Controllable andreev retroreflection and specular andreev reflection in a four-terminal graphene-superconductor hybrid system. *Phys. Rev. Lett.* **103**, 167003 (2009).

59. Y. Q. Sun, Y. Liu, S. Y. Hong, Z. Chen, M. Zhang, Y. W. Xie, Critical thickness in superconducting $LaAlO_3/KTaO_3$(111) heterostructures. *Phys. Rev. Lett.* **127**, 086804 (2021).

60. G. Herranz, G. Singh, N. Bergeal, A. Jouan, J. Lesueur, J. Gázquez, M. Varela, M. Scigaj, N. Dix, F. Sánchez, J. Fontcuberta, Engineering two-dimensional superconductivity and Rashba spin–orbit coupling in $LaAlO_3/SrTiO_3$ quantum wells by selective orbital occupancy. *Nat. Commun.* **6**, 6028 (2015).

61. Y. Li, H. Liu, H. Ji, C. Ji, S. Qi, X. Jiao, W. Dong, Y. Sun, W. Zhang, Z. Cui, M. Pan, N. Samarth, L. Wang, X. C. Xie, Q.-K. Xue, Y. Liu, J. Wang, High-temperature anomalous metal states in iron-based interface superconductors. *Phys. Rev. Lett.* **132**, 226003 (2024).

62. N. R. Werthamer, E. Helfand, P. C. Hohenberg, Temperature and purity dependence of the superconducting critical field, H_{c2}. III. Electron spin and spin-orbit effects. *Phys. Rev.* **147**, 295–302 (1966).

63. H. Eisaki, H. Takagi, R. J. Cava, B. Batlogg, J. J. Krajewski, W. F. Peck Jr., K. Mizuhashi, J. O. Lee, S. Uchida, Competition between magnetism and superconductivity in rare-earth nickel boride carbides. *Phys. Rev. B* **50**, 647–650 (1994).

64. N. Hadacek, M. Sanquer, J. C. Villégier, Double reentrant superconductor-insulator transition in thin TiN films. *Phys. Rev. B* **69**, 024505 (2004).

65. K. Maki, Critical fluctuation of order parameter in a superconductor I. *Prog. Theor. Phys.* **40**, 193–200 (1968).

66. R. S. Thompson, Microwave, flux flow, and fluctuation resistance of dirty type-II superconductors. *Phys. Rev. B* **1**, 327–333 (1970).

67. G. Bergmann, Weak localization in thin films: A time-of-flight experiment with conduction electrons. *Phys. Rep.* **107**, 1–58 (1984).

68. H. T. He, G. Wang, T. Zhang, I. K. Sou, G. K. L. Wong, J. N. Wang, H. Z. Lu, S. Q. Shen, F. C. Zhang, Impurity effect on weak antilocalization in the topological insulator Bi_2Te_3. *Phys. Rev. Lett.* **106**, 166805 (2011).

69. S. Mallik, G. C. Ménard, G. Saïz, H. Witt, J. Lesueur, A. Gloter, L. Benfatto, M. Bibes, N. Bergeal, Superfluid stiffness of a $KTaO_3$-based two-dimensional electron gas. *Nat. Commun.* **13**, 4625 (2022).

70. G. Zhang, L. Wang, J. Wang, G. Li, G. Huang, G. Yang, H. Xue, Z. Ning, Y. Wu, J.-P. Xu, Y. Song, Z. An, C. Zheng, J. Shen, J. Li, Y. Chen, W. Li, Spontaneous rotational symmetry breaking in $KTaO_3$ heterointerface superconductors. *Nat. Commun.* **14**, 3046 (2023).

71. K. Slevin, T. Ohtsuki, Corrections to scaling at the anderson transition. *Phys. Rev. Lett.* **82**, 382–385 (1999).
